# Photoacoustic polydopamine-indocyanine green (PDA-ICG) nanoprobe for detection of senescent cells

**DOI:** 10.1038/s41598-024-79667-7

**Published:** 2024-11-27

**Authors:** Muhamad Hartono, Andrew G. Baker, Thomas R. Else, Alexander S. Evtushenko, Sarah E. Bohndiek, Daniel Muñoz-Espín, Ljiljana Fruk

**Affiliations:** 1 Department of Chemical Engineering and Biotechnology, University of Cambridge, Cambridge, UK; 2 Early Cancer Institute, Department of Oncology, University of Cambridge, Cambridge, UK; 3 Cancer Research UK Cambridge Institute, University of Cambridge, Cambridge, UK; 4 Department of Physics, University of Cambridge, Cambridge, UK

**Keywords:** Senescence, Detection, Cancer, Polydopamine, ICG, Photoacoustic, Nanobiotechnology, Nanoscience and technology, Diseases, Cancer, Cancer imaging

## Abstract

Cellular senescence is considered an important tumour suppression mechanism in response to damage and oncogenic stress in early lesions. However, when senescent cells are not immune-cleared and persist in the tumour microenvironment, they can drive a variety of tumour-promoting activities, including cancer initiation, progression, and metastasis. Additionally, there is compelling evidence demonstrating a direct connection between chemo(radio)therapy-induced senescence and the development of drug resistance and cancer recurrence. Therefore, detection of senescent cells in tissues holds great promise for predicting cancer occurrence earlier, assessing tumour progression, aiding patient stratification and prognosis, and informing about the efficacy of potential senotherapies. However, effective detection of senescent cells is limited by lack of biomarkers and readout strategies suitable for in vivo clinical imaging. To this end, a nanoprobe composed of biocompatible polydopamine (PDA) nanoparticle doped with FDA-approved indocyanine green (ICG) dye, namely PDA-ICG, was designed as a contrast agent for senescence detection using photoacoustic imaging (PAI). In an in vitro model of chemotherapy-induced senescence, PDA-ICG nanoprobe showed an elevated uptake in senescent cells relative to cancer cells. In addition to its improved photostability, 2.5-fold enhancement in photoacoustic signal relative to ICG was observed. Collectively, the results indicate that the PDA-ICG nanoprobe has the potential to be used as a contrast agent for senescence detection of chemotherapy-induced senescence using PAI.

**Supplementary Information:**

The online version contains supplementary material available at 10.1038/s41598-024-79667-7.

## Introduction

Cellular senescence is a cell autonomous response to tissue damage, oncogenic stress and developmental cues^1^. The main function of senescence is to limit the expansion of damaged cells and to enable tissue repair^2^. In the senescence state, cells arrest their cell cycle and secrete a number of pro-inflammatory cytokines, chemokines and other factors collectively known as the senescence-associated secretory phenotype (SASP)^3^. In physiological conditions, the secretion of SASP recruits immune cells to clear senescent cells resulting in repair of the damaged tissue^1,3^. However, upon persistent damage such as those observed in diseased or pathological states, the removal of senescent cells becomes dysregulated, leading to the accumulation of these cells in the damaged tissues. This senescence accumulation has been linked to the development of age-related diseases, numerous cancers, and various chronic pathologies, including cardiovascular diseases, neurological disorders, fibrosis and diabetes^1,4,5^.

In the context of cancer, the presence of senescent cells serves a dual role^2,6^. On one hand, senescence plays a tumour suppression role in a cell autonomous manner by preventing the transformation of damaged/oncogenically-stressed cells into cancer cells^7,8^. However, in the tumour microenvironment, the presence of senescent cells has been demonstrated to promote metastasis^9–11^, angiogenesis^12^, epithelial-to-mesenchymal transitions (EMT)^11^, enhance stemness in cancer cells^13^, and contribute to a pro-tumorigenic microenvironment mediated through the inflammatory SASP^14^. In the context of treatment, it has been shown that the administration of chemotherapies and radiotherapies induces senescence at sublethal doses^9,15^. Such an increase in senescence has been demonstrated to promote tumour growth by fuelling the proliferation of bystander cells, facilitates cancer metastasis, and contributes to tumour relapse, ultimately resulting in poor patient survival^9,14,16,17^. Consequently, senescence has been recognized as one of the emerging hallmarks of cancer^18^.

In vivo detection of the senescence burden could aid the assessment of patient’s response to cancer treatment, risk stratification of cancer patients, adjustment of therapeutic protocols as well as early detection of cancer and administration of cancer preventative therapies in high-risk groups. Namely, cellular senescence is also a defining feature of premalignant lesions in multiple cancer types^8^, and monitoring the senescent burden would enable early therapeutic intervention and cancer prevention^19–21^. This is exemplified by the pharmacological eradication of senescent cells (senotherapies) that reduce tumour burden and extends survival^20,22^.

Unfortunately, longitudinal tracking of senescence via clinical imaging remains a formidable challenge^5,23,25^. Current methods for in vivo detection of senescence rely on invasive tissue biopsy followed by immunohistochemistry or fluorescent staining^5,23^. These strategies are mostly based on probes that monitor enzymatic activity of b-galactosidase enzyme present in lysosomal compartments within senescent cells. There are only a few imaging agents capable of tracking senescent lesions in mouse models, most which are based on modified and activatable fluorophores^26^. Positron emission tomography (PET) probes based on monitoring the activity of b-galactosidase enzyme are reported^27^, one of which is currently in Phase I clinical trial (NCT04536454)^28^. However, b-galactosidase is also present extensively in non-senescent highly secretory cell types such as macrophages and osteoclasts, limiting the specificity of these existing strategies^29,30^. In addition, fluorescence imaging is limited by penetration depth of the excitation light (fluorescence) making it unsuitable for in vivo human imaging. While PET imaging is limited by the use of radioactive labels and has high costs associated with instruments and infrastructure^24,31^.

**Fig. 1:**
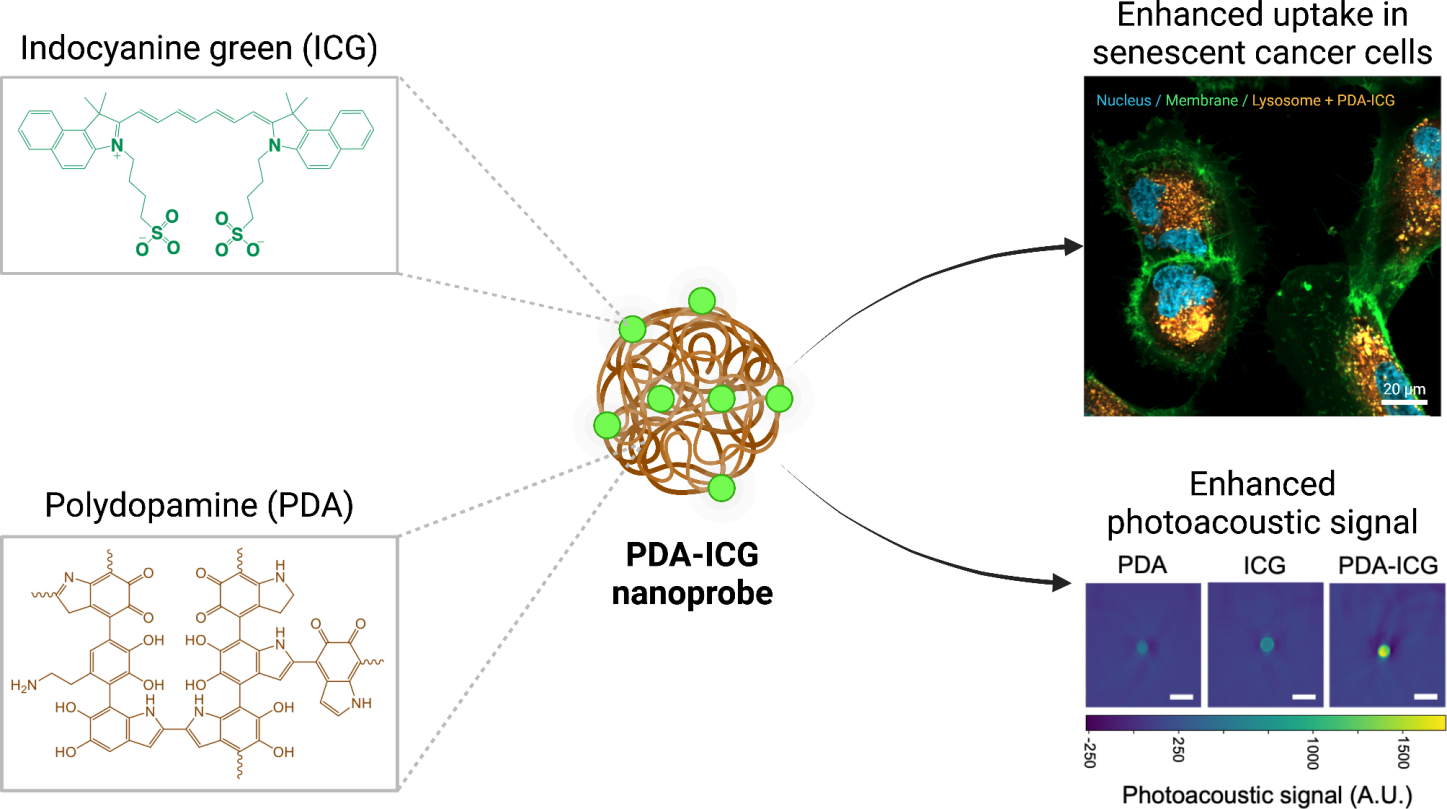
Schematic layout of present study. Photoacoustic-active PDA-ICG nanoprobe showing enhanced uptake in senescent cells. The nanoprobe was prepared by loading ICG dye into PDA nanoparticles.

Recognising the need for in vivo detection of the senescence burden, we have recently developed NanoJaggs, nanostructured probe made using indocyanine green (ICG) building block and suitable for in vivo detection using photoacoustic tomography^32^. To further improve photoacoustic signature and increase the versatility of functionalisation strategies, we present a design of nanoprobe composed of polydopamine (PDA) core doped with indocyanine green (ICG) dye to be used for photoacoustic imaging (PAI) of senescent cells (Fig. 1). PAI is a non-invasive imaging technique based on the detection of acoustic waves produced as short near infrared (NIR) laser pulses that are used to excite suitable endogenous (e.g. haemoglobin) or exogenous contrast agents^33^. Currently in clinical trials, PAI combines deep tissue penetration capabilities (up to 7 cm) with the high contrast of optical imaging, delivering superior spatial resolution when compared to ultrasound and fluorescence imaging, while avoiding infrastructure cost like that in PET imaging^34,35^. Furthermore, it can be utilised for both endogenous and exogenous contrast agents, enabling the construction of more comprehensive images through multiplexed imaging^28,36^.

In cancer research, PAI has already been explored for monitoring therapy response and diagnosing and characterizing primary tumors at a molecular level^37,38^. Several PAI contrast agents such as NanoJaggs, liposomal J-aggregates and gold nanoparticles have been reported^32,39,40^. Of note, ICG as a contrast agent is currently undergoing clinical trial for detecting lymph nodes in melanoma patients (NCT05467137). However, this dye has inherent potential issues, such as susceptibility to degradation and aggregation in aqueous solution, as well as poor photostability^40^. Additionally, it is difficult to functionalize ICG molecules with targeting or cargo ligands. Moreover, ICG is rapidly cleared from the body, with a short half-life of (2–4 min), which limits its ability to adequately label molecular targets^41,42^. Our PDA-ICG nanoprobe overcomes these limits by providing enhanced photoacoustic signal and improved stability. Furthermore, we also show a preferential uptake of our nanoprobe in senescent cells, while PDA core enabled further modification with range of functional moieties using mild chemical strategies^43^. Coupled with the excellent biocompatibility, we show that our probe can potentially be used an efficient PAI contrast agent to assess the burden of senescent cells in tissues.

## Results and discussion

### Synthesis and characterization of PDA-ICG nanoprobe

**Fig. 2:**
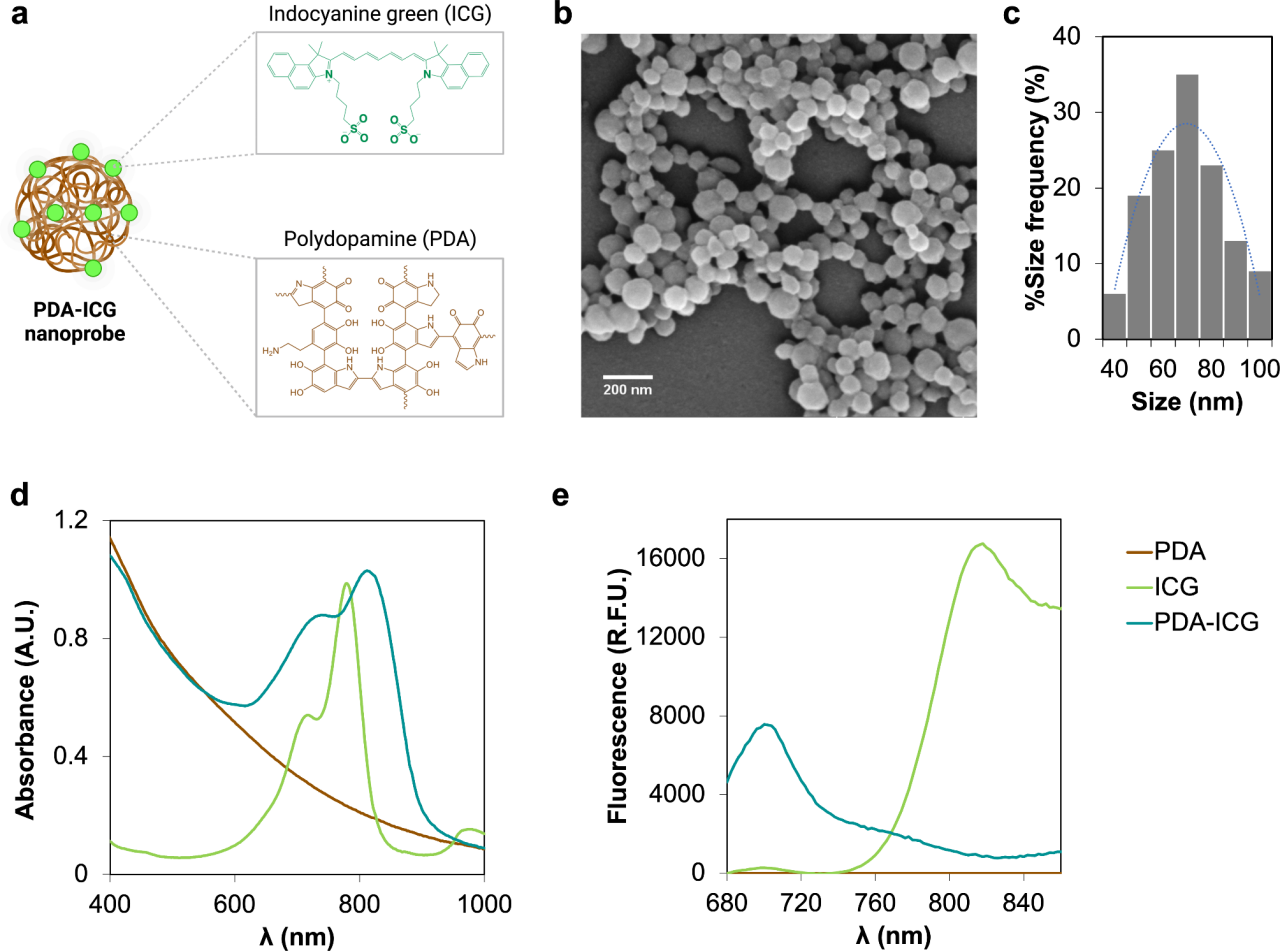
Synthesis and characterization of PDA-ICG nanoprobe. **a**. Schematic illustration of PDA-ICG molecular structures. **b**. SEM photograph showing spherical PDA-ICG, with corresponding (**c**) size distribution (*n* = 200 nanoparticles) with an average size of 75 ± 8 nm. **d.** Absorbance and (**e**) fluorescence emission spectra of bare PDA, ICG and PDA-ICG suspended in water (l_excitation_ = 640 nm). Compared to free ICG, the fluorescence emission of PDA-ICG shifts to lower wavelength (700 nm) and was quenched.

PDA-ICG nanoprobe was prepared in a two-step process that involved the preparation of PDA nanoparticles and doping of ICG dyes (Figs. 1 and 2a). Preparation of ICG-loaded PDA was previously reported resulting in larger (150 and 195 nm) nanoparticles^44,45^. However, unlike previous studies, our protocol involved heating the PDA and ICG mixture to enable self-assembly of ICG molecules on the surface of PDA to increase the ICG loading. Firstly, PDA nanoparticles were synthesized by self-polymerization of dopamine at pH 8.5 according to the reported protocol^46,47^. Subsequent addition of ICG to pre-made PDA at pH 3 and heating to 65°C yielded PDA-ICG nanoprobe. Spherical nanoparticles were obtained with an average size of 75 ± 8 nm according to SEM (Fig. 2b-c, Figure S1). After the addition of ICG, the average hydrodynamic size of nanoparticles increased from 134 nm (bare PDA NPs) to 198 nm, accompanied by the change in zeta potential from − 9 mV to -30 mV (Figure S2). Such decrease in zeta potential can be attributed to the presence of two negatively charged sulfonate groups of ICG, which also suggests the presence of ICG groups on the surface of PDA. This was further confirmed by FT-IR spectra (Figure S3) which contained both ICG and PDA peaks within the fingerprint region. Elemental and ICP analysis (Figure S4) estimated a loading percentage of ICG in PDA of 8% based on obtained sulfur content. An extinction coefficient of 27.334 (mg/mL)^−1^ cm^− 1^ was calculated for the nanoparticle employing UV-Vis spectroscopy (Figure S5).

To further confirm the presence of ICG, PDA-ICG nanoprobes were suspended in ethanol, which resulted in breakdown of the probe into ICG monomers and several ICG fragments as confirmed by mass spectrometry (Figure S6). Exposure to ethanol was used to break down the supramolecular ICG structure, which was confirmed by restoration of ICG fluorescence spectrum (Figure S7). Together, these findings suggested successful incorporation of self-assembled ICG on the surface of PDA nanoparticles.

Interestingly, a pH value of 3.0 was found to be the most suitable pH for preparation of PDA-ICG nanoprobe. When PDA NPs and ICG dye were mixed at pH 6.2-8, there were no significant changes in the hydrodynamic size and surface charge of PDA despite a longer mixing time (24 h vs. 4 h used for hybrid preparation at pH 3) (Table S1), indicating that the nanoprobe was not formed. At pH 3, the amino groups on PDA are protonated, resulting in an overall positive surface charge (Figure S8C) that allows for the initial electrostatic interaction of PDA with the negatively charged sulfonate groups of ICG, which further facilitates hydrophobic interactions and π-π stacking resulting in stable hybrid formation.

The non-covalent interactions between PDA and ICG influence the optical properties of the resulting nanoprobes. PDA broadly absorbs in the UV region whereas ICG has a characteristic absorbance peak at 780 nm and a shoulder peak at 690 nm, corresponding to its monomer and dimer conformation, respectively (Fig. 2d). In contrast to bare PDA, PDA-ICG spectrum shows a peak in the NIR region (812 nm). When heated, ICG dye forms a packed J-aggregate structure *via* π-π stacking and electrostatic interactions it shows a red-shifted absorbance peak in the NIR region (895 nm)^48,49^. When a mixture of PDA and ICG is not heated a PDA-ICG product with an absorbance peak at 800 nm is obtained (Figure S8A). Similar results were reported previously and indicate the adsorption of cyanine dyes onto the nanoparticle core^50^. The heating process is likely to induce self-assembly of ICG molecules on the surface of PDA nanoparticle, possibly resembling J-aggregate-like structures^32^. As a result, we obtained a higher ICG loading (8%) using our heating protocol compared to the previously reported values (2–4%). Self-assembly of ICG could also explain the red-shift of approximately Dl = 30 nm in PDA-ICG when compared to free ICG. The PDA-ICG fluorescence (Fig. 2e) is also altered compared to free ICG, with quenching of ICG emission at 810 nm and the emergence of a lower intensity emission peak at 700 nm. This finding is to be expected since PDA has been shown to be an effective fluorescence quencher^51^.

**Fig. 3:**
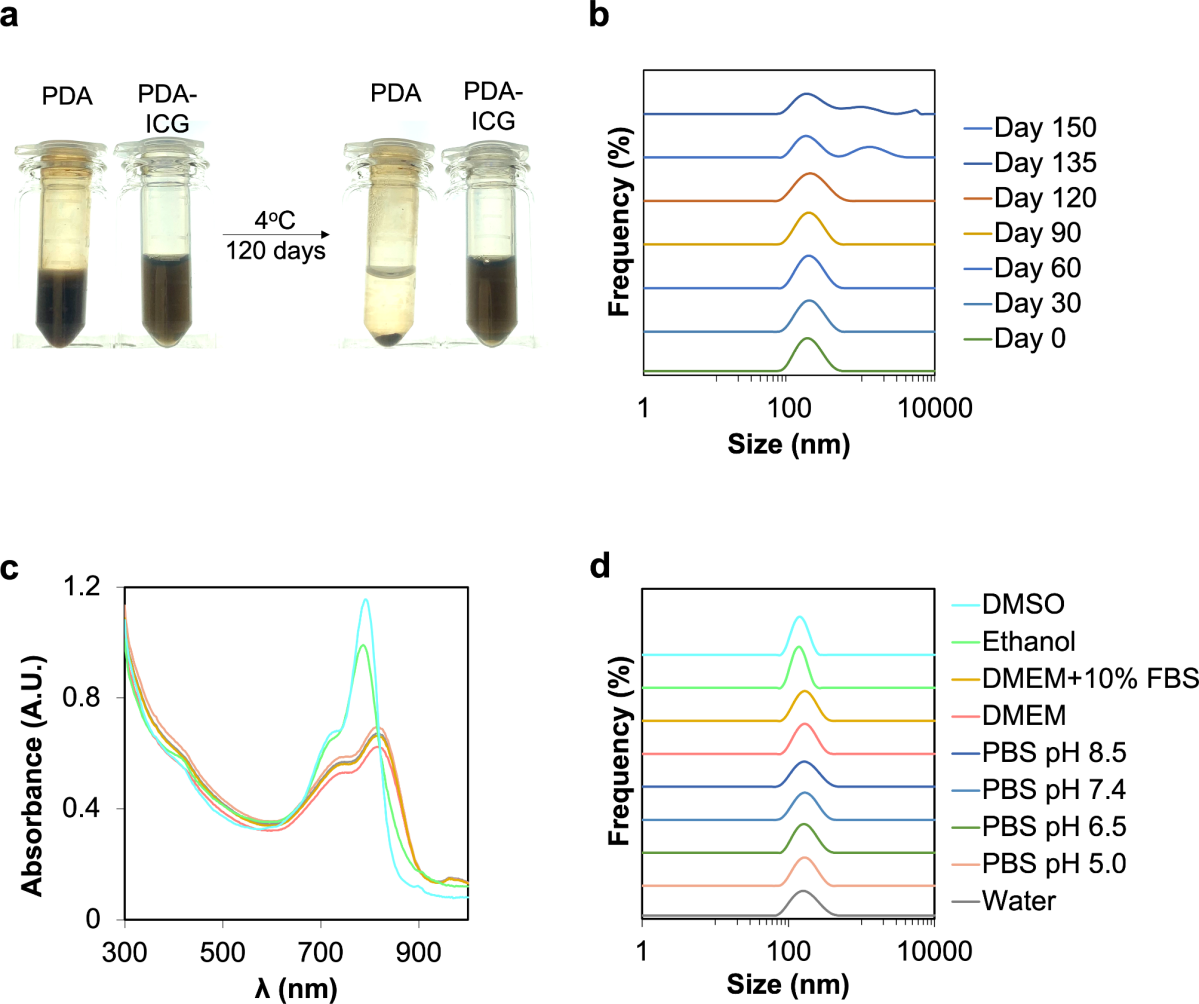
PDA-ICG exhibits excellent colloidal and long-term stability. **a**. Images of PDA and PDA-ICG suspended in water at the same concentration (0.5 mg/ml), before and after being stored at 4°C for 120 days. **b**. Corresponding DLS measurements of PDA-ICG in water over different period of storage. Conjugating PDA with ICG improves nanoparticles colloidal stability. Colloidal stability of PDA-ICG suspended in deionized water, biological media and organic solvents measured with UV-Vis spectroscopy (**c**) and DLS (**d**). Measurement in biological media were taken after 24 h incubation at 37°C, while in other media were taken 5 min after the nanoprobe was suspended. PDA-ICG was stable in biological media but were broken apart in organic solvents.

Next, we wanted to explore the colloidal stability of PDA-ICG. This was done by incubating the aqueous and buffer suspension of the nanoprobe at different temperatures. Remarkably, conjugation of ICG to PDA has marked positive effect on the stability of the suspension, with PDA-ICG remaining stable in deionized water at 4°C for up to 120 days (Fig. 3a-b, Figure S9B). In contrast, bare PDA completely precipitated out of the suspension after this time, while ICG was stable only for 3 days in dark, after which it degraded completely^52^. PDA-ICG could also be stored as a dry powder enabling long-term storage after lyophilization in presence of maltose (30% weight) as cryo-protectant. The resulting dark green solid could be easily re-suspended without causing significant changes in its shape and hydrodynamic size (Figure S9C).

Given that we wanted to employ PDA-ICG in cell culture studies, we also tested its colloidal stability in biological media. PDA-ICG remained stable after 72 h incubation at 37°C in cell culture media containing serum (DMEM with 0–10% FBS) and in physiological buffer (phosphate buffer saline, PBS pH 5.5–8.5), with no significant changes both in size (no aggregation or protein corona formation) and absorbance (Fig. 3c-d). However, the changes in absorbance spectra in DMSO and ethanol suggest the disassembly of PDA-ICG in organic solvents due to the disruption of electrostatic interactions and π-π stacking interactions. Nevertheless, when incubated in relevant biological media, 79 ± 5% of ICG remained incorporated in PDA (Figure S10), suggesting that the nanoprobe remains stable over extended period and could be used for subsequent cell studies.

Another advantage of using PDA-ICG system is the ability to use the PDA core for further functionalization, which allows the addition of desirable moieties either for specific tissue targeting or delivery of therapeutic cargo^47^. As a proof of concept, we explored EDC/NHS amide coupling to allow binding of arginine amino acid to the free amines of PDA (Figure S11) and employed Michael addition to add a TAMRA-labelled positively charged peptide to the quinone or indole components of PDA (Figure S12). We have previously used arginine and histidine labelled PDA for delivery of DNA cargo, while addition of peptides has both the targeting and therapeutic advantages^43^.

Successful preparation of functionalized PDA-ICG is confirmed by spectroscopic analysis, as well as DLS, zeta potential and elemental analysis in Table S2. Arginine-functionalized PDA-ICG (PDA-ICG-arginine) can be prepared by first attaching arginine to PDA core followed by ICG incubation (Figure S13C-E, Table S2). Similarly, peptide-functionalized PDA-ICG (PDA-peptide-ICG) was prepared by first modifying PDA core with peptide followed by heat-mediated addition of ICG (Figure S13F-I).

### Senescent cells internalize more PDA-ICG nanoprobes than cancer cells

**Fig. 4:**
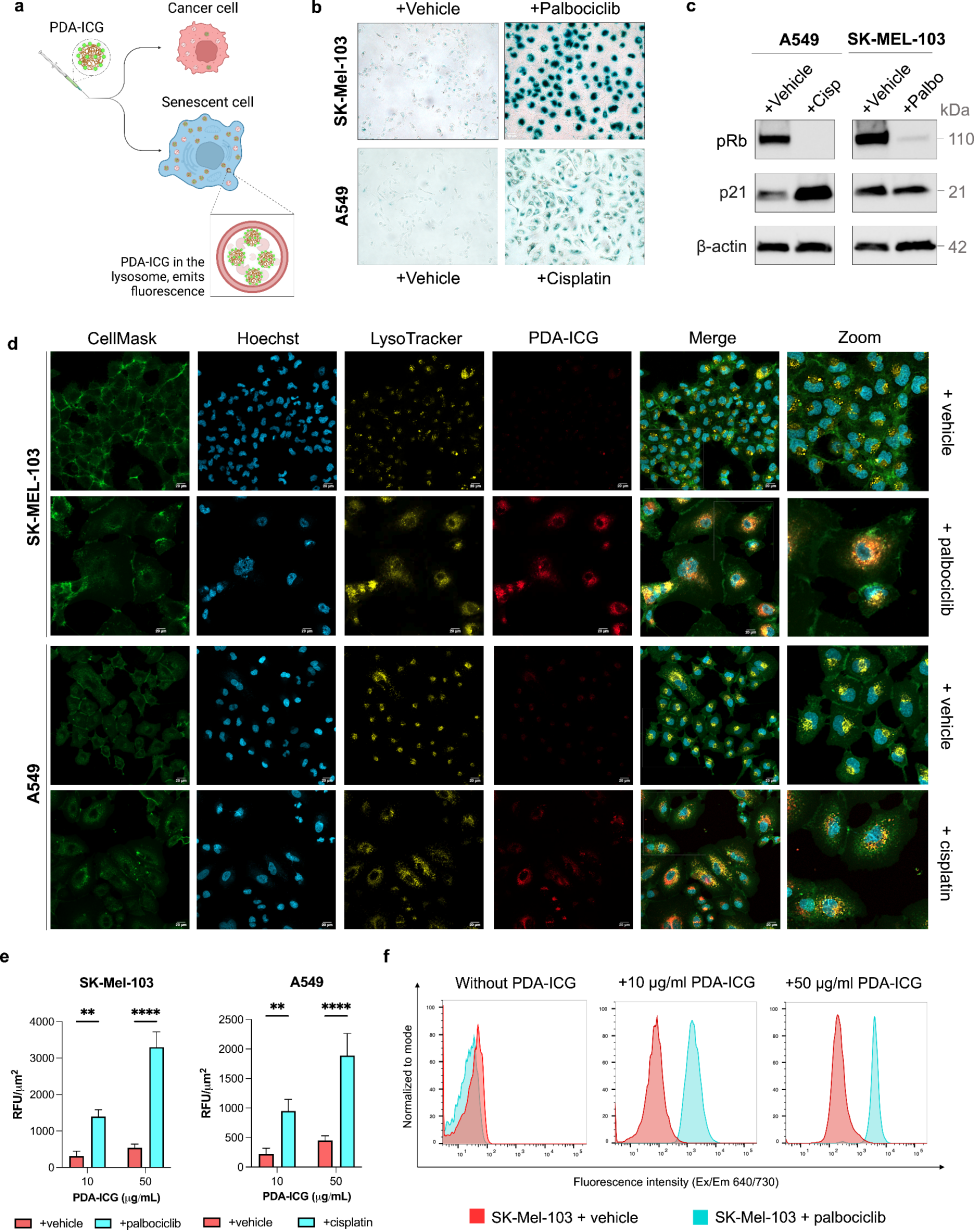
Study of cellular uptake and intracellular localization of PDA-ICG nanoprobe on SK-MEL-103 and A549 cells using confocal fluorescence microscopy and flow cytometry. **a**. Schematic illustration showing the cellular uptake of PDA-ICG by either cancer cells or senescent cells. Cells were first validated for senescence 10 days following treatments with either chemotherapeutic drugs or vehicles. **b**. Representative images of b-galactosidase staining of fixed control (vehicle-treated) cells, palbociclib-treated SK-MEL-103 cells and cisplatin-treated A549 cells. **c**. Western blot analysis of the expression of relevant senescence markers: phosphorylated retinoblastoma (pRb), and cell cycle inhibitor p21 with β-actin as reference. **d**. Confocal microcopy images of cells following 24-h incubation with 10 µg/ml PDA-ICG nanoparticles. Cell membranes, nucleus and lysosomes were stained with CellMask (green), Hoechst 33,342 (blue) and LysoTracker (yellow). PDA-ICG was observed in the Alexa Fluor 700 channel (red). Scale bar = 20 μm. **e**. Corresponding fluorescence intensity for nanoprobe-treated cells, showing senescent cells having higher PDA-ICG fluorescence intensity compared to cancer cells (mean ± SD, *n* = 3, ** for *p* ≤ 0.01, **** for *p* < 0.0001). **f**. Histogram showing mean fluorescence values of SK-MEL-103 cells after incubation with PDA-ICG; PDA-ICG fluorescence was measured from the Alexa Fluor 700 channel that corresponded to the fluorescence of PDA-ICG nanoparticles.

Senescent cells are characterized by the significantly higher number and activity of lysosomes relative to cancer and healthy cells possibly due to accumulation of damage products, altered metabolic activity, and/or a result of the arrest in cell division^53–55^. Interestingly, ICG dye has been shown to accumulate in the lysosomes, possibly via clathrin-dependent endocytosis^56,57^. We have also recently shown that the uptake of small organic nanoparticles, NanoJaggs, entirely composed of ICG, is significantly increased for senescent cells^32^. Taking this into account, we hypothesized that PDA-ICG would exhibit an enhanced uptake in senescent cells compared to cancer cells (Fig. 4a).

To investigate our hypothesis, we first established an in vitro model of therapy-induced senescence by subjecting two cancer cell lines, A549 (human adenocarcinoma) and SK-MEL-103 (human melanoma). Cells were treated with varying durations and concentrations of chemotherapeutic drugs cisplatin and Palbociclib until optimal concentrations for senescence inductions were achieved. Senescence of the cells was confirmed by detection of several hallmarks of senescence^58^. b-galactosidase staining confirmed a significant increase in senescence-associated b-galactosidase-positive cells for all chemotherapeutic-treated cells compared to vehicle-treated (control) cells (Fig. 4b, Figure S14A). Additionally, drug-treated cells stopped proliferating, suggesting cell cycle arrest that is a characteristic of senescence, whereas control cells showed a normal cell growth pattern (Figure S14B). We further validated the upregulation of several senescence markers at the protein level (Fig. 4c), including the decrease in phosphorylated retinoblastoma (pRb) protein, and increase in cell cycle inhibitor p21 for cisplatin treatment due to the DNA-damaging mechanism. Of note, the other chemotherapeutic employed, Palbociclib, directly inhibits cyclin-dependent kinases therefore the senescence program does not involve upregulation of p21. The RT-qPCR analyses (Figure S14C) also confirmed increased expression of *CDKN1A* and *GLB1* genes and the downregulation of *LMNB1* gene, both of which are hallmarks of senescence. Altogether, these results validated that A549 and SK-MEL-103 cells effectively became senescent cells upon drug treatments.

To assess the biocompatibility of PDA-ICG nanoprobe a standard MTS assay on both the cancer and senescent A549 and SK-MEL-103 cells was performed. Each cell line was treated with PDA-ICG over a range of concentrations and incubated for 72 h. It is evident from Figure S15 that PDA-ICG nanoprobe did not significantly affect the viability of all cell lines for at least up to 100 µg ml^− 1^, suggesting that the nanoprobe can safely be used in further cell studies within this concentration range.

After completing this characterization, the cellular uptake of PDA-ICG by cancer cells versus senescent cells was assessed using fluorescence confocal imaging. Although the fluorescence of PDA-ICG at 810 nm is quenched, PDA-ICG emits quantifiable fluorescence at around 700–730 nm (Fig. 2c). Cell nuclei, lysosome and cell membranes were labeled to distinguish intracellular PDA-ICG from those adhered to the cell surface. PDA-ICG nanoprobes were internalized in the cells after addition of 10 µg/mL PDA-ICG (Fig. 4d, for images with a higher magnification, see Figure S16; for images without PDA-ICG and with 50 µg/mL PDA-ICG, see Figure S17). Senescent cells have flattened morphology in vitro with larger surface area compared to cancer cells. Because of this, we normalized the fluorescence signal to the area of the cells. As shown in Fig. 4e, incubation of PDA-ICG nanoprobe in senescent cells resulted in an enhanced fluorescence (ca. 6-fold for senescent SK-MEL-103 cells and 4.2-fold for senescent A549) relative to cancer cells, suggesting that significantly more PDA-ICG nanoprobe was internalized within drug-treated (senescent) cells relative to cancer cells. Of note, these values are comparable to the reported uptake from previous studies of different senescent probes using the same cell lines^32,59^. In addition, as previously shown by us, ICG uptake does not significantly change when control and senescent cells are compared^32^. This increase in cellular uptake could also be observed under brightfield light microscopy (Figure S22). The higher uptake of PDA-ICG nanoparticles in senescent cells relative to cancer cells can be explained by the elevated number of and enlarged lysosomes in senescent cells^53–55^. Additionally, senescent cells have also been shown to have an enhanced uptake of sulfonate-containing indocyanine green particles^32^.

The co-localization of the LysoTracker and the PDA-ICG signal indicates that the probe is localized within lysosomes. Flow cytometry was also performed to quantify the cellular uptake of PDA-ICG. Upon treating both senescent and cancer cells populations with the same concentration of PDA-ICG, the fluorescence intensity in the senescent cell population was significantly higher than in the cancer cell population (Fig. 4f and Figure S18). This observation provides further evidence that the PDA-ICG nanoprobe exhibits a higher accumulation in senescent cells, indicating its potential for distinguishing between cancer and senescent cell populations. Interestingly, when ICG is loaded into PDA nanoparticles, prepared using protocols from previous studies which does not involve heating (refer to Figure S8B for the corresponding absorbance spectrum)^44,45^ showed an overall cellular uptake that is similar to the background (without particles), with similar uptake in both senescent and non-senescent cells (Figure S19) in contrast to PDA-ICG nanoprobe. This is possibly because of the lower ICG loading in these ICG-loaded PDA nanoparticles, which resulted in lower stability in cell media. This finding highlights the importance of ICG in PDA-ICG nanoprobe for improving the cellular uptake.

To demonstrate the modifiability of PDA-ICG, we functionalized it with a fluorophore-labelled peptide. Cell studies were also performed with this peptide modified PDA-ICG (PDA-Peptide-ICG) to further validate the peptide addition does not affect the ICG-nanoparticle formation and uptake. We find nearly identical cellular uptake profiles using flow cytometry (Figure S20) as well as colocalization with the lysosomes of the senescent cells (Figure S21). These initial studies have indicated huge potential of hybrid PDA-ICG system for design for theranostic probes, and further studies are currently ongoing exploring the delivery of biologics to senescent cell.

As we and others already reported earlier, increased number of lysosomes in senescent vs. healthy cells can account for increased uptake of nanoparticles observed^53–55^. In addition, ICG has been shown to accumulate in the lysosomes possibly via active clathrin-dependent endocytosis^56,57,60^. Further to this, similar nanoparticle system composed of a dimer of ICG has also demonstrated reliance on this pathway for cell uptake within senescent cells^32^.

### PDA-ICG nanoprobes show enhanced photoacoustic signal while being more photostable than ICG

**Fig. 5:**
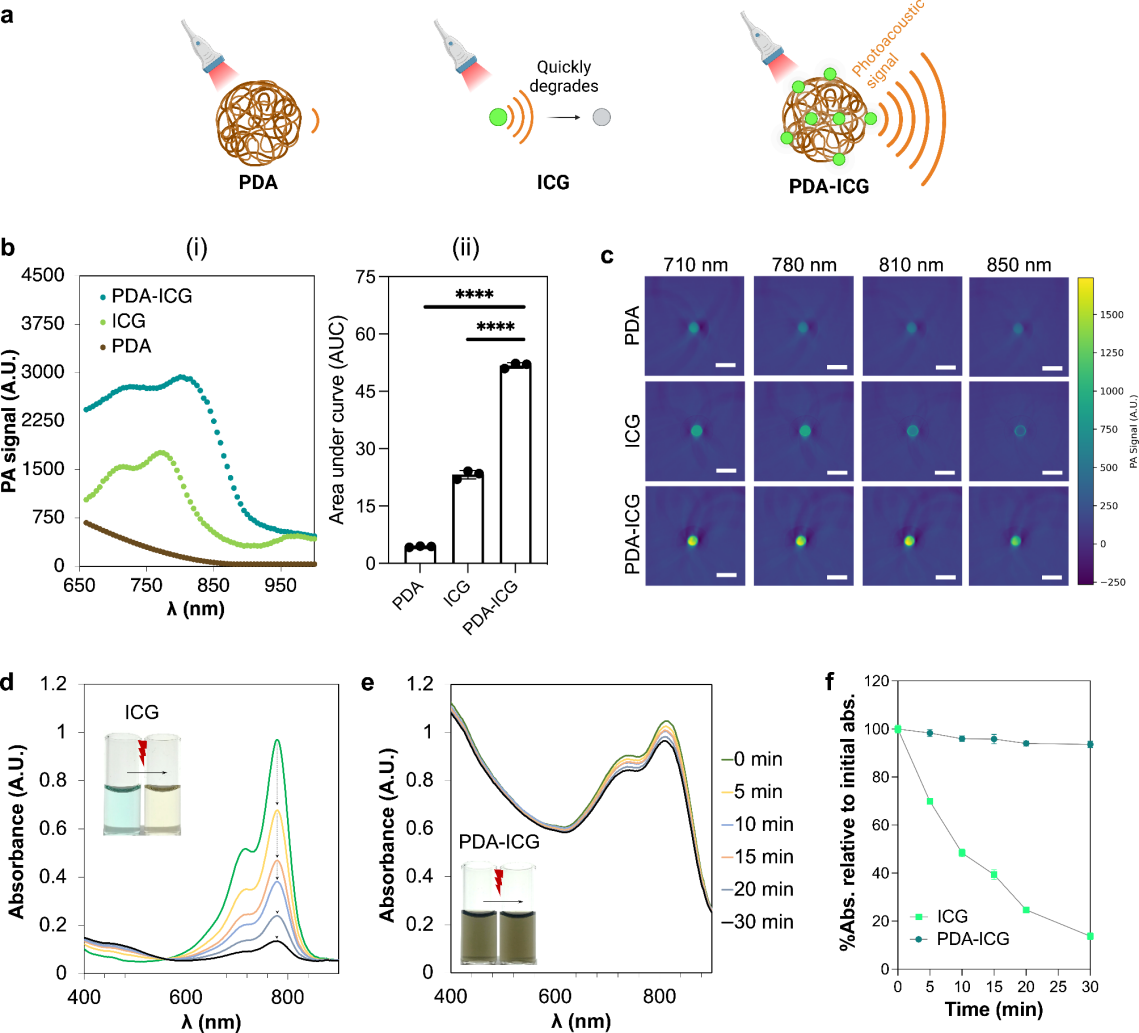
Photoacoustic properties and photostability of PDA-ICG nanoprobe. **a)** Schematic illustration of the comparison among photoacoustic signals generated by PDA, ICG and PDA-ICG. **b)** (i) Photoacoustic signal in phantoms generated by bare PDA, free ICG and PDA-ICG (left). (ii). Area under the curve (AUC) measured from (i) that represents the integrated photoacoustic mean pixel intensities (mean ± SD, *n* = 3, **** for *p* < 0.0001, ordinary one-way ANOVA). **c**) Phantom photoacoustic images of bare PDA, free ICG and PDA-ICG at different excitation wavelengths. Absorbance spectra of free ICG (**d**) and PDA-ICG (**e**) taken immediately after irradiation with a 740 nm laser. Insets are photographs of ICG and PDA-ICG solution taken before and after laser irradiation. **f**. Percentage of absorbance over time relative to the initial absorbance of ICG versus PDA-ICG, wherein the absorbance of ICG is constantly declining over time.

It has been shown previously that photoacoustic signal of a dye can be enhanced when its fluorescence is quenched^61–65^, due to a higher light-to-thermal conversion efficiency^66^. Because PDA is an effective fluorescence quencher (Fig. 2d), we hypothesized that PDA-ICG probe should exhibit enhanced photoacoustic signal compared to ICG. To assess the photoacoustic properties of PDA-ICG nanoprobe and the controls, all materials were encapsulated in cylindrical tissue-mimicking phantoms with defined optical properties closely mimicking the optical properties of biological tissue (Fig. 5a)^67^. Individual phantoms were then imaged using a commercial photoacoustic tomography system and analyzed as described previously^64^. To compare the photoacoustic efficiency, the same absorbance of PDA, ICG and PDA-ICG at their respective maximum absorbance wavelengths was used since photoacoustic signal depends on the amount of light absorbed by materials (Figure S23).

Figure 5b (i) shows the photoacoustic spectra of PDA, ICG and PDA-ICG that exhibited signal peaks that corresponded to their respective absorbance peaks. Bare PDA nanoparticles displayed broad melanin-like photoacoustic signals^68^. However, since the peak was not within NIR region, PDA alone cannot be utilized as a photoacoustic probe. The area under the curve (AUC), that reflects the integrated mean pixel intensities, indicated an average of 2.5-fold increase in photoacoustic signal generation efficiency for PDA-ICG compared to free ICG (Fig. 5b, ii). The enhancement is evident also in the spectrally reconstructed photoacoustic images of the channel containing PDA-ICG, which appeared brighter compared to the channels containing either free ICG or bare PDA (Fig. 5c) at various excitation wavelengths. Notably, the peak of photoacoustic signal is at a slightly lower wavelength than our previously developed NanoJagg probe (895 nm)^32^ while the photoacoustic signal enhancement is comparable to that of NanoJagg.

Notably, PDA-ICG exhibited not only enhanced photoacoustic signal compared to ICG, but also demonstrated improved photostability. One of the primary limitations of using small-molecule dyes like ICG as contrast agent is their rapid degradation. When exposed to light, their degradation is significantly accelerated, which restricts the imaging time to only a few minutes after injection. As shown in Fig. 5d and **f**, after irradiation with 740 nm laser, ICG bleached within minutes and its absorbance was decreased by 50% after only 10 min, and by 90% after 30 min, accompanied by visible color change from light green to light brown. In contrast, under the same experimental conditions, the absorbance of PDA-ICG remained unchanged (Fig. 5e-f), indicating that PDA-ICG exhibited higher photostability compared to ICG dyes. The enhanced photostability of PDA-ICG can be attributed to several factors. Firstly, PDA can absorb some of the light thereby reducing the amount of light that reaches ICG dyes. Secondly, PDA contains hydroquinone groups which are efficient radical scavengers, effectively neutralizing singlet oxygen species and preventing the photobleaching of ICG.

Such increased photostability together with biocompatibility and enhanced photoacoustic signal makes PDA-ICG system excellently suited for in vivo biodistribution and imaging studies, which we are currently in the planning phase using relevant models of therapy-induced senescence.

## Conclusion

PDA-ICG nanoprobe described in this study has huge potential as contrast agent for photoacoustic imaging of senescent cells. Composed of the core polydopamine (PDA) nanoparticles and self-assembled indocyanine dye (ICG) structures, PDA-ICG formed upon heating of PDA and ICG mixture. The prepared nanoprobe demonstrates remarkable colloidal stability in a wide range of biological media and can be stored for months both in aqueous solution and in powder form.

We observed that our PDA-ICG nanoprobe preferentially accumulates in chemotherapy-induced senescent cells, most likely due to the combination of lysosome localization and an active, ICG-mediated cell uptake. Such senescence selective uptake together with enhanced photoacoustic signal makes PDA-ICG ideal contrast agent for photoacoustic imaging, which is increasingly employed as non-invasive and cost-effective alternative to other in vivo imaging strategies. The addition of ICG is shown not only to have significant positive effect on colloidal stability but contributes to 2.5-fold enhancement of photoacoustic (PA) signal compared to ICG. Furthermore, unlike ICG dye, PDA-ICG probe is not rapidly degraded under conditions used to obtain PA signal, indicating huge potential of this ICG hybrid to be used *for in vivo* monitoring which requires longer circulation and imaging times.

Further studies will focus on shedding more light on the specific uptake of sub-100 nm ICG containing nanostructures to senescent cells, and the application of PDA-ICG for PA imaging using in vivo murine models. However, we believe that present study clearly highlights a significant potential of our hybrid nanoparticles to be used as photoacoustic probes for detection of senescent cells. Considering the proven role of senescence in initiation, progression and recurrence of cancer and multiple age-related diseases, the development of selective multifunctional nanostructures for the detection of the senescent burden is one of the strategies that will bring us closer to efficient, precision medicine strategies. In addition, we demonstrate that due to the presence of modifiable PDA core, hybrid nanoparticles can be designed that contain various covalently attached moieties opening a route not only to imaging but also delivery of therapeutic cargo, which would significantly improve the specificity of therapeutic interventions for senescence cells.

## Materials and methods

### Materials

All materials were purchased in their highest available purity. Indocyanine green (ICG, IR-125) was purchased from Acros Organics. Dopamine hydrochloride, fetal bovine serum (FBS), Trizma base, Dulbecco’s modified eagle medium (DMEM), ethanol, dimethyl sulphoxide (DMSO), phosphate-buffered saline (PBS), L-arginine, 1-ethyl-3-(3-dimethylaminopropyl) carbodiimide (EDC) and N-hydroxysuccinimide (NHS) were purchased from Sigma-Aldrich. Ammonium hydroxide (NH_4_OH) was purchased from Acros Organics. Reagents for MTS assay was purchased from Promega, USA. Cisplatin was purchased from Stratech while Palbociclib (PD0332991 isethionate) was purchased from Pfizer Inc. TAMRA-labelled peptide was purchased from Bioserv UK.

### Synthesis and characterization of PDA-ICG nanoprobe

#### PDA synthesis

The protocol to prepare PDA nanoparticles was adapted from Chen et al.^47^. Trizma base (45 mg) was dissolved in 5 mL deionized water and then added to a solution of 15 mL DMSO and 45 mL deionized water, followed by 30-min magnetic stirring. Subsequently, dopamine (46 mg) was added to the reaction. The mixture was left overnight under stirring (500 rpm). Purification of nanoparticles was achieved by centrifugation at 4,000 xg (4°C) for 15 min, followed by centrifugation of the obtained supernatant at 18,000 xg for 20 min. The resulting pellet was washed with deionized water thrice.

#### PDA-ICG synthesis

PDA-ICG was prepared by adding ICG (9 mg) to a 10-mL suspension of PDA nanoparticles in deionized water (0.125 mg/mL) with pH 3.0, adjusted by adding HCl. The mixture was sonicated for 10 min, following which was heated to 65°C and left to stir (500 rpm) for 4 h. The resulting particles were purified by centrifugation (like PDA synthesis) and washing with deionized water five times.

#### PDA-arginine synthesis

Arginine-functionalized PDA (PDA-arginine) was prepared through amide coupling between the carboxylic acid of arginine and primary amines of PDA. PDA (4 mg) was suspended in 30 mL of NH_4_OH solution. An aqueous 10-mL solution of L-arginine (20 mg), EDC (76 mg) and NHS (46 mg) was stirred for 30 min. The arginine solution was then added dropwise to the PDA nanoparticle suspension followed by vigorous stirring overnight. The final concentration of NH_4_OH was 10 mM. The resulting particles were purified by centrifugation (like PDA synthesis) and washing with deionized water five times.

#### PDA-peptide synthesis

Peptide-functionalized PDA (PDA-peptide) was prepared through Michael addition between the cysteine thiols and unsaturated carbon bonds of PDA. PDA (500 µg) was mixed with TAMRA-labelled peptide (500 µg) in 3 mL of 10 mM Tris buffer pH 8.5. Nanoparticles were obtained as pellets by centrifuging the mixture at 10,000 rpm for 10 min and washing with ethanol and deionized water consecutively.

#### ICG release

PDA-ICG (0.1 mg/mL) was firstly dissolved in phosphate buffer saline (PBS) at pH 7.4 and DMEM supplied with 10% FBS at 37°C. ICG release in these media was then investigated by collecting the released ICG by centrifugation for different periods of time and then the absorbance was measured using UV-Vis spectroscopy.

#### Photostability

PDA-ICG and ICG, both suspended in deionized water, were separately added to a 96-well plate. Concentrations were adjusted to give an absorbance value of 1 A.U. Samples were then irradiated with a 740 nm laser (54 mW/cm^2^) and the absorbance spectra were recorded over time.

#### Colloidal and long-term stability

The colloidal stability of PDA-ICG was evaluated in deionized water, PBS pH = 5.5–8.5, cell growing media (DMEM and DMEM + 10% FBS). Suspensions of these media (1 mL) containing 0.1 mg/mL of PDA-ICG were incubated for 72 h at 37°C. Following incubation, the hydrodynamic size, zeta potential and UV-Vis measurements of the suspensions were recorded. The same experiment was done for PDA-ICG suspended in ethanol and DMSO, except measurements were taken 5 min after suspension. For long term stability, a suspension of PDA-ICG (0.1 mg/mL) in 1-mL deionized water was kept for 150 days in a 4°C (protected from light).

#### Characterization

DLS and zeta potential measurements were performed using a Zetasizer Nano Range instrument (Malvern Analytical). SEM was performed using a TESCAN MIRA3 FEG-SEM. ImageJ software was used to calculate the diameter of 150–200 individual nanoparticles. UV-Vis spectra were recorded with an Agilent Cary 300 UV-Vis spectrophotometer. Fluorescence emission spectra were recorded using a Varian Cary Eclipse Fluorescence Spectrophotometer. Fourier transform infrared spectroscopy (FT-IR) spectra were recorded using a Bruker Tensor 27 FT-IR spectrometer with samples pressed into KBr pellets. CHN combustion was performed on a CE-440 Elemental Analyser from Exeter Analytical, Inc. (combustion temperature 975°C). ICP analysis was performed on an iCAP7400 Duo ICP spectrometer from Thermo Fisher Scientific.

#### Photoacoustic measurements

Photoacoustic measurements were performed using a commercial photoacoustic tomography system (inVision256-TF; iThera Medical GmbH). Briefly, the system uses a tunable (660–1300 nm) optical parametric oscillator pumped by a nanosecond pulsed Nd: YAG laser operating at 10 Hz repetition rate for signal excitation. As the detector, a transducer array (5 MHz center frequency, 60% bandwidth, toroidal focusing) was used. Tissue mimicking phantoms were used to closely mimic the optical and acoustic properties of biological tissues. ICG, PDA-ICG and PDA were encapsulated separately inside thin walled optically transparent tubes located at 1 cm depth along the center of the phantom, which was placed inside the imaging chamber of the photoacoustic system. Photoacoustic signals were acquired at 710, 780, 810 and 850 nm excitation wavelengths. Mean pixel intensity (MPI) values were then obtained from a region of interest drawn within the thin-walled plastic straw (tubes) and then averaged from five different scan positions. Photoacoustic signal generation efficiency was obtained by normalizing the absorbance of ICG and PDA-ICG to 1 A.U. to give fair comparison since the generated photoacoustic signal depends on the amount of light absorbed by materials. The absorbance of PDA was matched to the PDA absorbance that gave 1 A.U. in PDA-ICG.

### In vitro evaluation of PDA-ICG nanoprobe

#### Cell lines and growth conditions

Human melanoma (SK-MEL-103) and human lung adenocarcinoma (A549) cell lines were purchased from American Type Culture Collection (ATCC). All cell lines were grown in a complete growth medium (DMEM supplemented with 10% fetal bovine serum (FBS)). These cells were cultured in a humidified incubator at 37°C with 5% CO_2_. Experiments were formed using cells at passage number between 5 and 18.

#### Cytotoxicity studies

Cellular toxicity of PDA-ICG on the viability of drug-treated (senescent) and vehicle-treated (non-senescent) SK-MEL-103 and A549 cells was evaluated using a standard MTS assay. Cells (2000 cells/well) were seeded into clear 96-well plates in 100 µL complete growth medium and cultured for 24 h at 37°C. Following which, the cells were treated with varying concentrations of PDA-ICG (0.001–500 µg/mL) suspended in complete growth media containing 0.4% PBS. MTS assay was performed according to the manufacturer’s instruction. After further 72 h incubation, 20 µL of CelTiter 96^®^ One Solution was added into each well and incubated for 2 h at 37°C. Subsequently, the absorbance of each well was measured at 490 nm using a Spark plate reader (TECAN). Control measurements, to account for potential sample interferences with MTS reagent, included negative control (cells only with DMEM), cells with DMEM containing 0.4% PBS, cell-free DMEM + 10% FBS (blank) and cell-free nanoprobe dilution in culture media. All experiments were conducted in biological triplicates. Cell viability was calculated as a percentage according to the following equation:

$$\:Cell\:viability=\frac{Absorbance\:of\:treated\:cells-Absorbance\:of\:blank}{Absorbance\:of\:control-Absorbance\:of\:blank}\:x\:100\%$$

#### Senescence induction

For chemotherapy-induced senescence, cisplatin was reconstituted in sterile PBS while palbociclib was prepared in DMSO. A549 cells were treated with 15 µM cisplatin for 10 days. SK-MEL-103 cells were treated with 5 µM Palbociclib for 10 days. These cells were used for experiments immediately after drug removal.

#### β-galactosidase staining

After 10 days under drug treatment, cells were washed thoroughly with pre-warmed PBS and then fixed and stained for senescence-associated β-galactosidase activity using the Senescence β-galactosidase Staining Kit (Cell Signaling) as per manufacturer’s instructions. After staining, cells were washed with PBS and then imaged using an Olympus Compact Brightfield Modular Microscope (Life Technologies). Total number of senescence-associated-β-galactosidase positive cells (blue colored) was counted to determine the proportion of senescent cells after drug treatments.

#### **RNA extraction**,** cDNA synthesis and quantitative real-time PCR**

RNA from cells was extracted using the RNeasy Mini Kit (Qiagen) and resuspended in RNase-free water. Complementary DNA (cDNA) was synthesized from the extracted RNA with the High-Capacity RNA-to-cDNA Kit (Thermo Fisher Scientific) using a total of 500 ng RNA per reaction. The levels of senescence-associated genes (*LMNB1*,* GLB1*,* CDKN1A*) were measured by RT-qPCR of the synthesized cDNA performed on a Quantstudio 1 Real-Time PCR instrument (Applied Biosystems) using Luna Universal qPCR Master Mix (New England Biolabs) according to the manufacturer’s instructions. Relative quantification was performed using the 2^−ΔΔCt^ method. A list of primers used to amplify senescence-associated genes are detailed in Table S2.

#### Western blot

To extract proteins from cells, cells were lysed using RIPA buffer (Sigma) supplemented with phosphatase inhibitors (PhosSTOP™ EASYpak Phosphatase Inhibitors Cocktail, Roche) and protease inhibitors (cOmplete™ Protease Inhibitor Cocktail, Roche). Proteins were then quantified, separated by SDS-PAGE and transferred to polyvinylidene difluoride (PVDF) membranes (Millipore) following standard protocols. Membranes were then blotted with primary antibodies against β-actin, p21, and pRb. Following overnight incubation at 4°C, membranes were rinsed and incubated with secondary HRP-conjugated AffiniPure antibodies for 1 h at room temperature. Subsequently, the membranes were incubated with Enhanced Chemiluminescence Detection solution (Amersham) and then imaged with a ChemiDoc imager (Bio-Rad) at automatic exposure times. Details on the antibodies used are available on Table S4 and Table S5.

#### Confocal imaging

Cells were seeded into a glass bottom dish (MatTek Life Science, US) (150,000 cells/well for non-senescent (control) cancer cells, 200,000 cells/well for senescent cells) and incubated at 37°C for 24 h, following which the cells were treated with different concentrations of PDA-ICG for another 24 h at 37°C. After 3 washed with PBS the cells were stained with CellMask™ Green (Thermo Fisher) plasma membrane stain, nucleus stain Hoechst 33,342 (Thermo Fisher) and lysosome stain LysoTracker Red (Thermo Fisher) according to the manufacturer’s instructions. Cells were then washed gently with PBS three times, following which the cells were imaged using Axio Observer Z1 LSM 800 (Zeiss) confocal microscope. Image acquisition and processing were performed using Zen software (Zeiss).

#### Flow cytometry

Cells seeded at a 2 × 10^5^ cells/well density in a 6-well plate were cultured for 24 h. Subsequently, the cells were treated with 10 and 50 µg/mL PDA-ICG prepared in the culture media and incubated for 24 h. After being incubated with PDA-ICG, cells were washed with PBS three times to remove the residual nanoprobes both in culture media and on the cell surfaces. Cells were then detached with 0.25 mL TrypLE (Thermo Fisher) and centrifuged for 5 min at 300 xg, 4°C. The cell pellets were then resuspended in 1 mL of FACS buffer (PBS with 4% FBS), into which 0.1 µg/mL DAPI dye was added. Cells were kept on ice (4°C) until flow cytometry experiments. Flow cytometry was performed on a Canto II flow cytometer (BD Biosciences) using 355, 488 and 640 lasers. As many as 10,000 events were acquired for each sample. Data analysis was performed using FlowJo software (version 10.2). In short, the population of live cells was gated in a plot of FSC versus DAPI channel, then cell debris and doublets were excluded from the live single-cell population by gating the plot of FSC versus SSC. Finally, a histogram from the 640/730 nm channel, which corresponded to the fluorescence emission from PDA-ICG, for the live, single-cell population was obtained and analyzed. This population of cells was deemed to internalize the nanoprobe.

#### Statistical analysis

Experiments in this study were independently repeated at least in triplicates and all data were presented as mean ± standard deviation. The GraphPad Prism 10 software (GraphPad Software, USA) was used to perform all statistical analysis; student -test or 2Way ANOVA depending on the dataset. Significance levels are defined as the following: not significant (ns) for *p* > 0.05, * for *p* ≤ 0.05, ** for *p* ≤ 0.01, *** for *p* < 0.001, and **** for *p* < 0.0001.

## Electronic supplementary material

Below is the link to the electronic supplementary material.

Supplementary Material 1

## Data Availability

Data is provided within the manuscript and supplementary information files. Original Western blot file for Fig. 4c is included in Suplementary File Fig. S24.
