# Supplementary material for: Photoacoustic polydopamine-indocyanine green (PDA-ICG) nanoprobe for detection of senescent cells
Source: Sci Rep. 2024 Nov 27;14:29506. doi: 10.1038/s41598-024-79667-7 (PMC11603024; doi:10.1038/s41598-024-79667-7)
Supplement: Supplementary file 1 — Supplementary Material 1 [file 41598_2024_79667_MOESM1_ESM.pdf]

Electronic Supplementary Information

Photoacoustic Polydopamine-Indocyanine Green (PDA-ICG) Nanoprobe for Detection of Senescent Cells

Muhamad Hartono[1†], Andrew Baker[1,2†], Tom Else[3,4], Alexander Evtushenko[1], Sarah Bohndiek[3,4], Daniel Muñoz-Espín[2], Ljiljana Fruk[1*]

[1]Department of Chemical Engineering and Biotechnology, University of Cambridge, Cambridge, UK.
[2]Early Cancer Institute, Department of Oncology, University of Cambridge, Cambridge, UK.
[3]Cancer Research UK Cambridge Institute, University of Cambridge, Cambridge UK.
[4]Department of Physics, University of Cambridge, Cambridge, UK.
*Corresponding author: lf389@cam.ac.uk
†These authors contributed equally.

Table of Contents

1. Characterization of PDA-ICG nanoprobe 3
2. Functionalization of PDA and loading of ICG into pre-functionalized PDA ..13
3. In vitro cell studies..16

1. Characterization of PDA-ICG nanoprobe

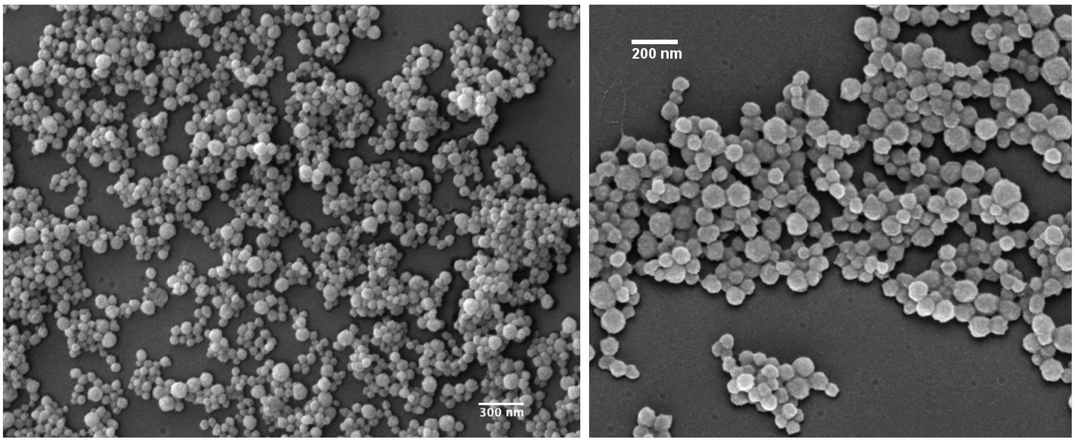

Figure S1. SEM images of PDA-ICG nanoprobe.

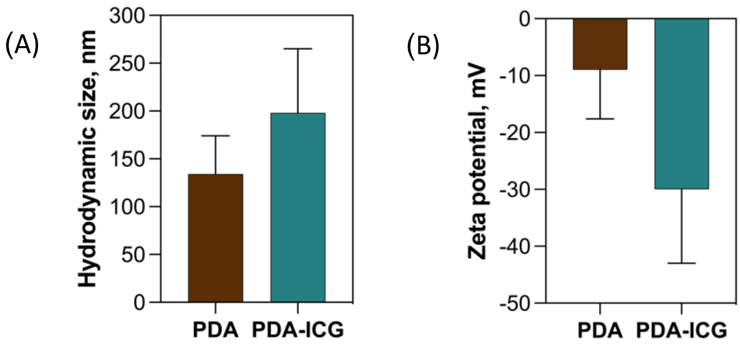

Figure S2. Hydrodynamic size (A) and surface charge (B) of PDA and PDA-ICG. Samples were suspended in deionized water at 0.01 mg/ml.

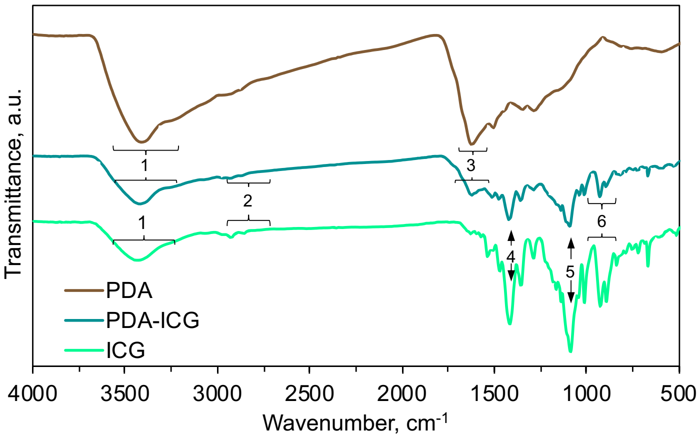

Peak	
**1** (3300-3700 cm^{-1})	N-H and O-H stretching vibrations
**2** (2800-300 cm^{-1})	C-H stretching vibrations
**3** (1385 cm^{-1})	C-OH phenolic bending
**4** (1335-1372 cm^{-1})	S=O stretching in the sulfonate groups
**5** (1085 cm^{-1})	=C-H binding vibrational modes
**6** (925-1010 cm^{-1})	=C-H binding vibrational modes

Figure S3. **FT-IR spectra of PDA, ICG and PDA-ICG.** Structural features were attributed to peaks based on literature.[33–35] PDA-ICG spectrum showed characteristic PDA bands (peak **1** and peak **3)**, as well as fingerprint bands for ICG (peaks **2**, **4**, **5** and **6)**.

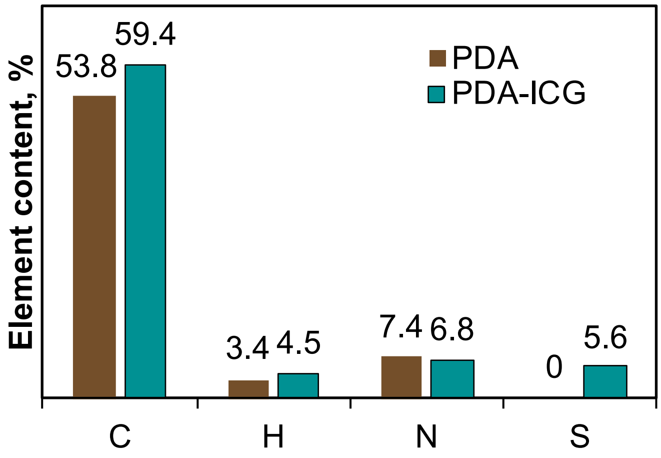

Figure S4. Elemental analysis of PDA and PDA-ICG. PDA-ICG contained sulphur (S) atoms from ICG while bare PDA did not. The C, H and N content was obtained from CHN combustion analysis while S content was obtained from ICP by dissolving PDA-ICG in nitric acid-water solution.

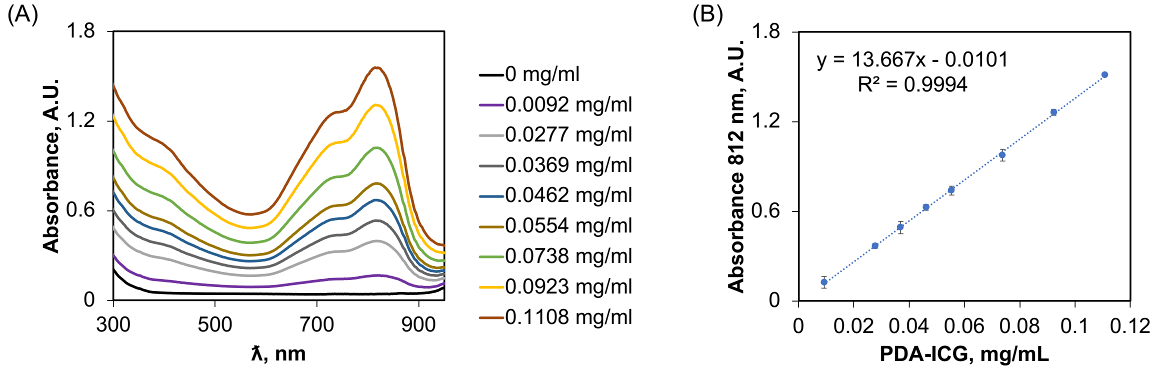

Figure S5. Extinction coefficient measurement of PDA-ICG. UV-Vis spectroscopy of PDA-ICG suspension in water under various concentration. Path length of 5 mm was used. Using Lambert-Beer's law, an extinction coefficient (ε) of 27.334 $(mg/mL)^{-1}$ cm^{-1} was calculated.

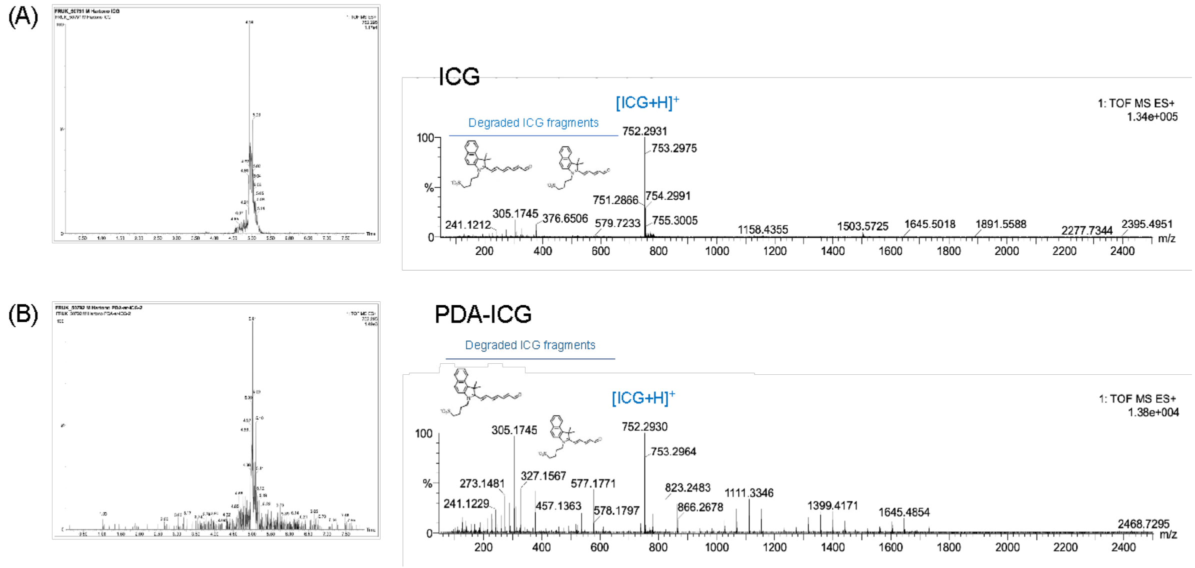

Figure S6. LC-MS spectra of ICG and PDA-ICG. Samples were suspended in water:acetonitrile solution (1:1 v/v). Peaks corresponding to ICG monomer and fragmented products of ICG were identified in PDA-ICG, confirming the presence of ICG in PDA-ICG. HRMS: calculated for $C_{43}H_{47}N_2O_6S_2^-$ $(M+H)^+$: Mass predicted: 752.29; Found: 752.2930.

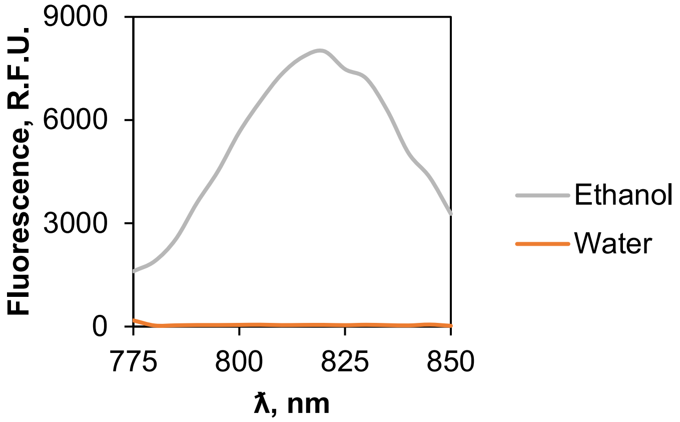

Figure S7. Fluorescence spectrum of PDA-ICG in water and ethanol. When PDA-ICG nanoparticles were suspended in ethanol, a new emission peak around 810 nm appeared that corresponded to the ICG monomers.

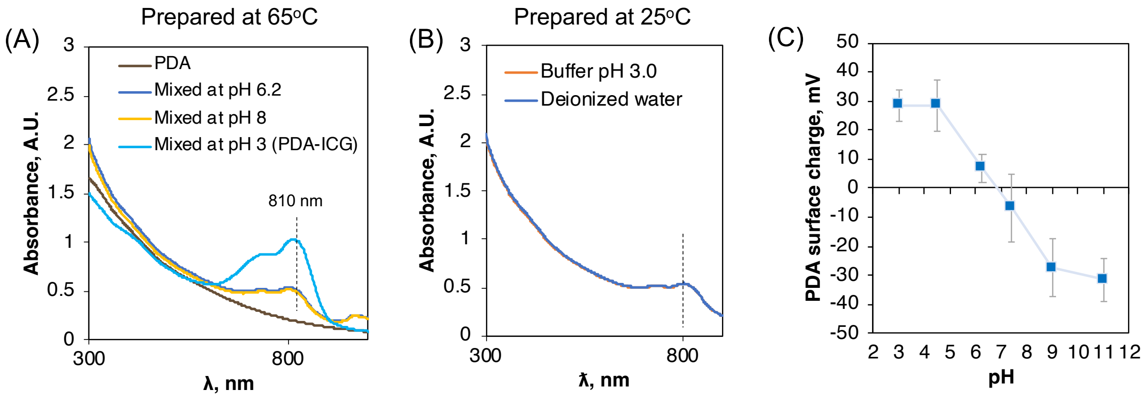

Figure S8. Analysis of PDA-ICG preparation under different reaction conditions. (A, B) UV-Vis spectroscopy of products after mixing PDA and ICG in various conditions. (B) is referred to as ICG-loaded PDA. (C) Zeta potential of PDA nanoparticles measured at different pH (mean ± s.d., n = 3).

(A)

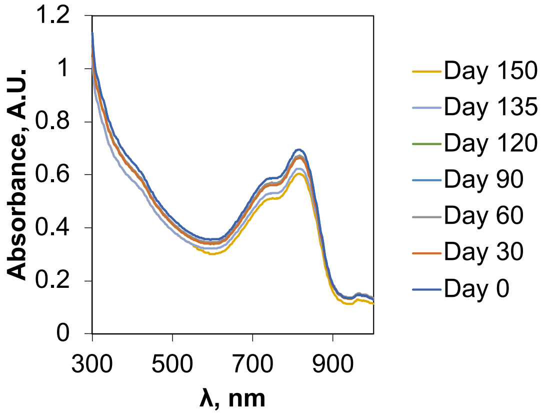

(B)

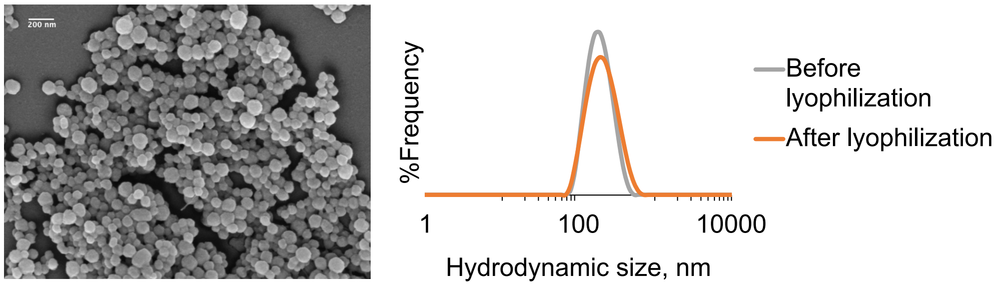

Figure S9. Analysis of long-term stability of PDA-ICG. Samples were suspended in deionized water at 4°C using UV-Vis spectroscopy (A). PDA-ICG was colloidally stable as a suspension up to 120 days; aggregation was observed after 135 days incubation. B. SEM image (top) and hydrodynamic size of PDA-ICG after lyophilization (30% maltose was used as cryo-preservant). Maltose was removed from the nanoparticles by three cycles of centrifugation and washing with deionized water.

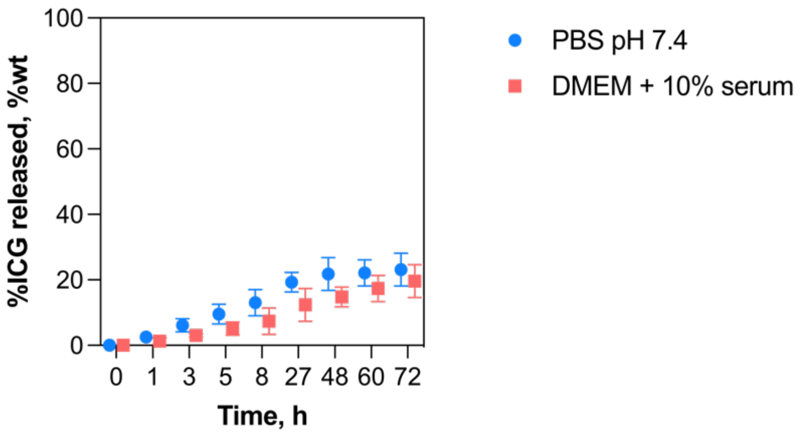

Figure S10. ICG release from PDA-ICG. Samples were incubated for 72 h at 37ºC in relevant biological media (PBS pH 7.4 and DMEM supplemented with 10% FBS serum). Data is presented as mean ± s.d. (n = 3).

2. Functionalization of PDA and loading of ICG into pre-functionalized PDA

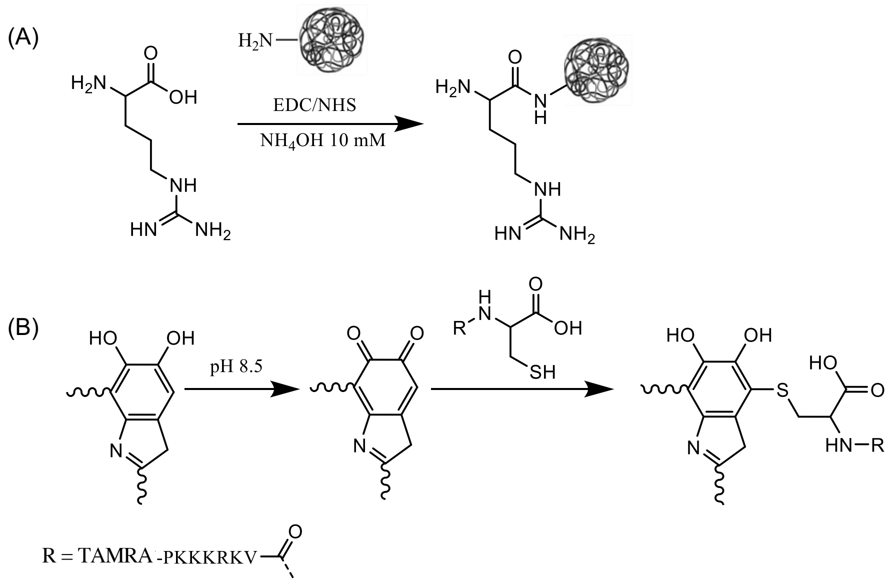

Figure S11. Reaction scheme for functionalization of PDA nanoparticles, with (A) arginine via amide coupling and (B) Peptide-TAMRA via Michael addition.

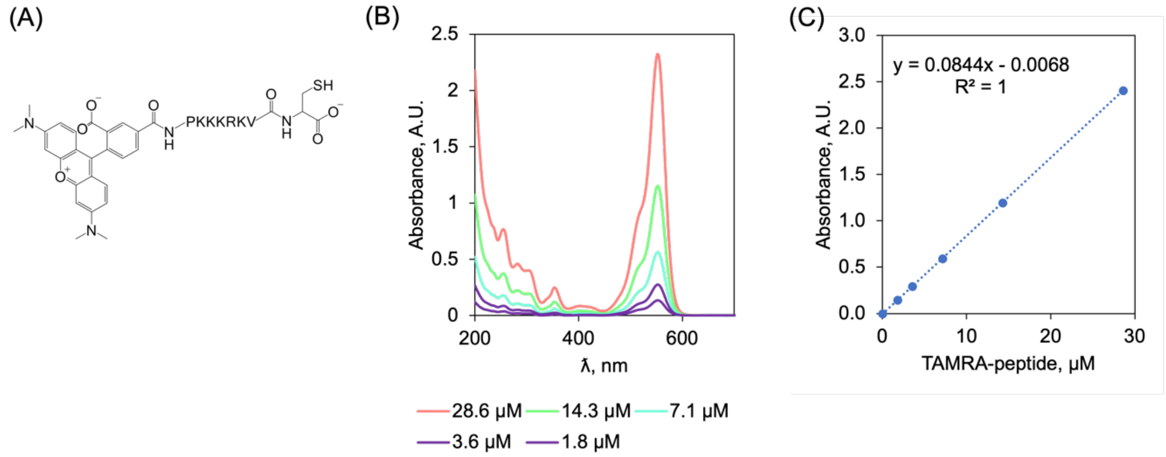

Figure S12. **Structure and absorbance of TAMRA-labelled peptide**. (A) Chemical structure of TAMRA-peptide. (B) Absorbance of TAMRA-peptide at different concentrations in water. (C) Calibration standard curve relating the concentration of TAMRA-peptide versus its absorbance (mean ± s.d., n = 3).

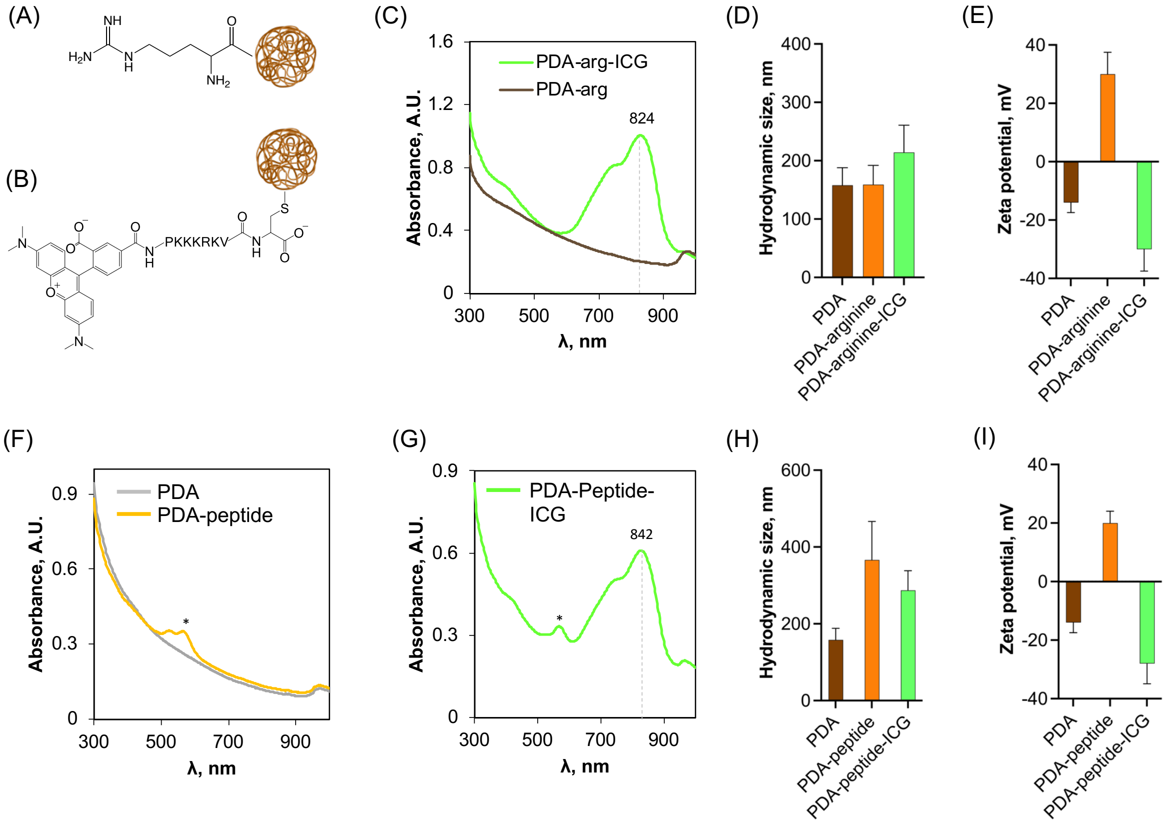

Figure S13. Loading of ICG into pre-functionalized PDA nanoparticles. Illustration of (**a**) PDA-argine and (**b**) PDA-peptide. **c**. Absorbance spectra of PDA-arg and PDA-arg-ICG. **d**. hydrodynamic size of PDA, PDA-arg, and PDA-arg-ICG by DLS. **e**. Zeta Potential of nanoparticles PDA, PDA-Arg, and PDA-Arg-ICG. **f**. Absorbance spectra of PDA-peptide. **g.** Absorbance of PDA-Peptide-ICG. **h**. Hydrodynamic size of PDA, PDA-Peptide, and PDA-Peptide-ICG by DLS. **i**. Zeta Potential of nanoparticles PDA, PDA-Peptide, and PDA-Peptide-ICG. Data in D, E, H and I are presented as mean ± s.d., n = 3.

3. *In vitro* cell studies

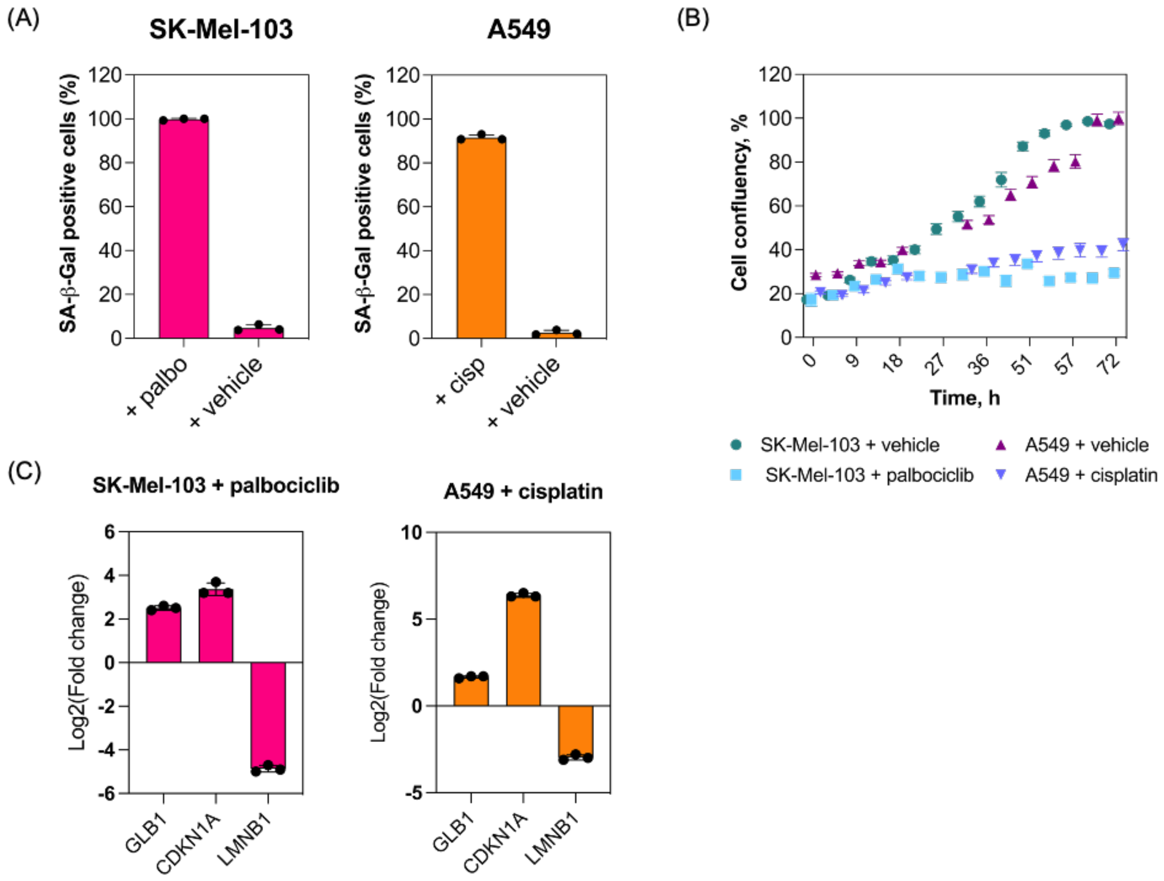

Figure S14. Additional validation of the induction of cellular senescence in cell lines 10 days following treatments with either chemotherapeutic drugs or vehicles. Quantification of β-galactosidase-positive cells (A) as shown in Figure 3b (mean ± s.d., n = 3). (B) Cell confluency over time of vehicle-treated and drug-treated cells, showing that drug-treated cells have an arrested growth (*n* = 3). (C) Fold change gene expression levels of relevant senescence markers in senescent cells relative to vehicle-treated cells using *ACTB* as reference (mean ± s.d., n = 3).

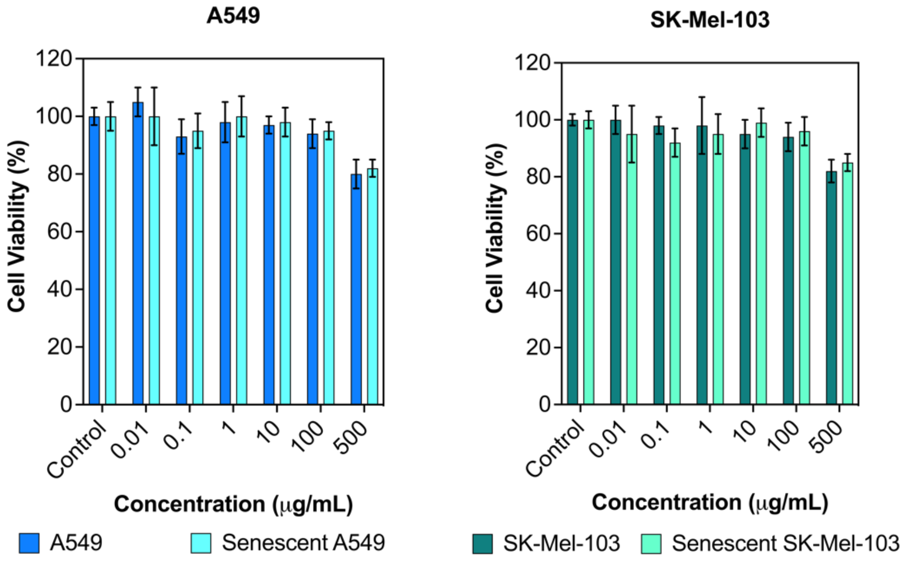

Figure S15. Assessment of *in vitro* cytotoxicity of PDA-ICG on SK-Mel-103 and A549 cell lines after 72 h incubation determined by MTS assay. Data are expressed as the mean ± SD obtained from triplicate experiments.

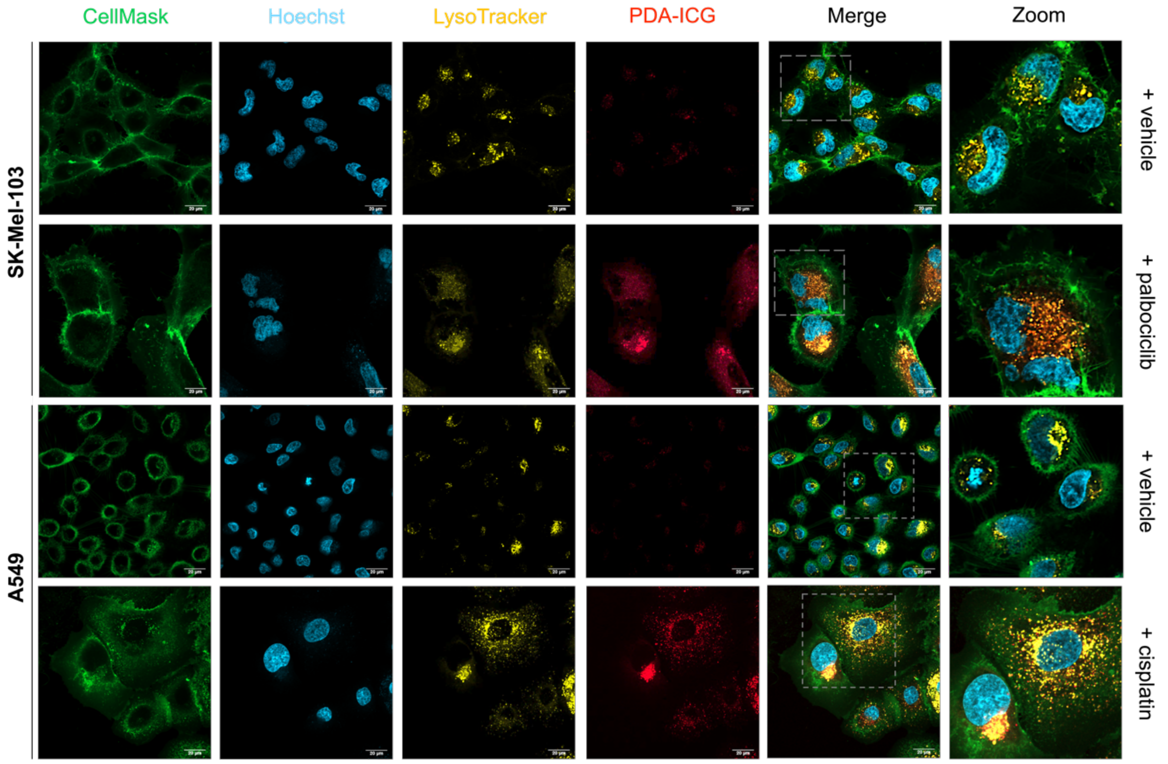

Figure S16. Higher resolution confocal microcopy images of cells following 24-h incubation with 10 μg/ml PDA-ICG nanoparticles. Cell membranes, nucleus and lysosomes were stained with CellMask (green), Hoechst 33342 (blue) and LysoTracker (yellow). PDA-ICG was observed in the Alexa Fluor 700 channel (red). Scale bar = 20 μm.

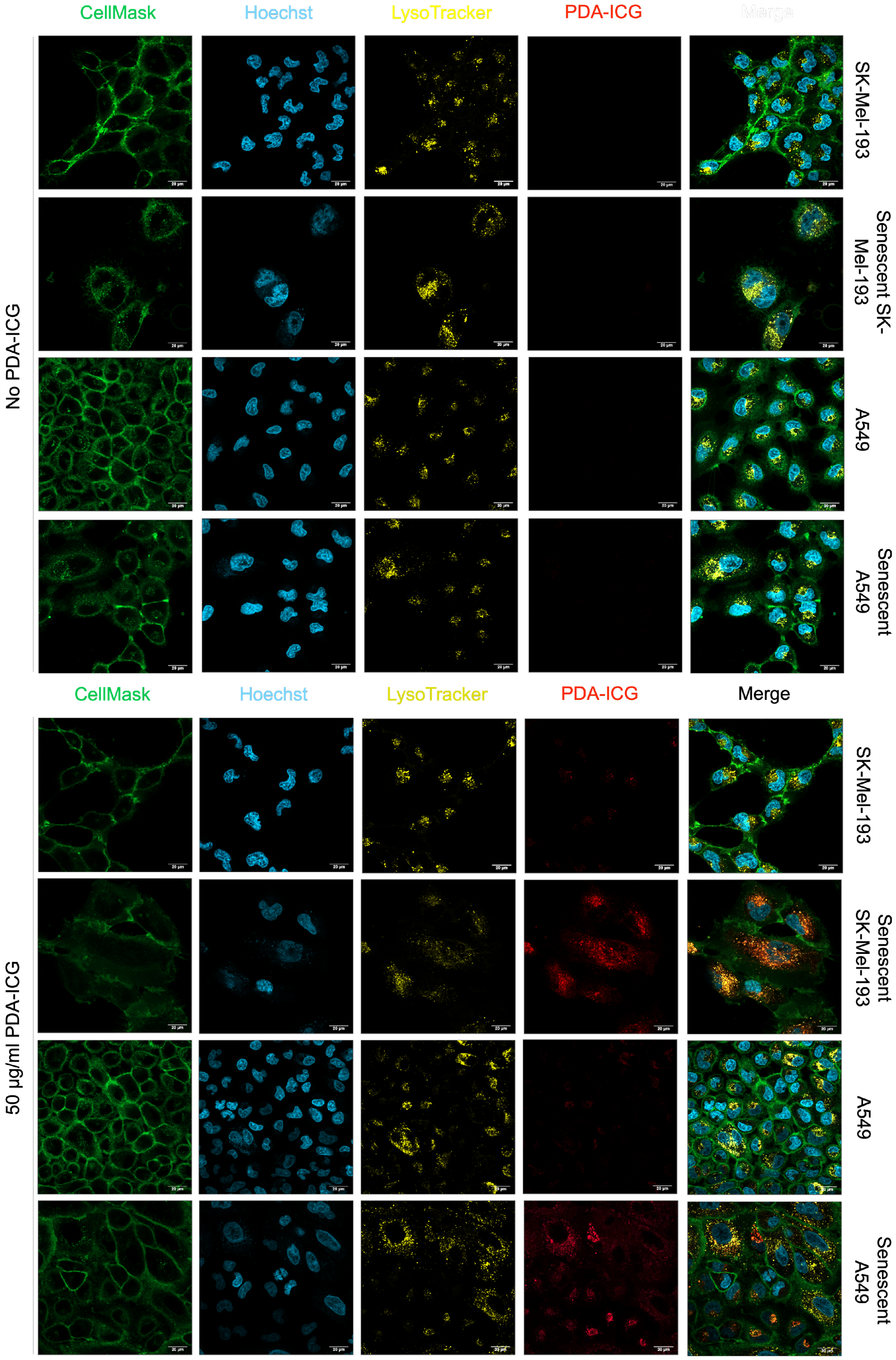
CellMask
Hoechst
LysoTracker
PDA-ICG
Merge
SK-Mel-193
Senescent SK-Mel-193
A549
Senescent A549
No PDA-ICG
50 µg/ml PDA-ICG

Figure S17. Fluorescence confocal images of A549 and SK-Mel-103 cells after incubation with PBS (vehicle, control) and 50 μg/mL PDA-ICG for 24 h. Cells were incubated with CellMask (green), Hoechst 33342 (blue) and LysoTracker Green (yellow) to stain cell membrane, nuclei, and lysosome. Alexa Fluor 700 channel (red) captured the fluorescence of PDA-ICG.

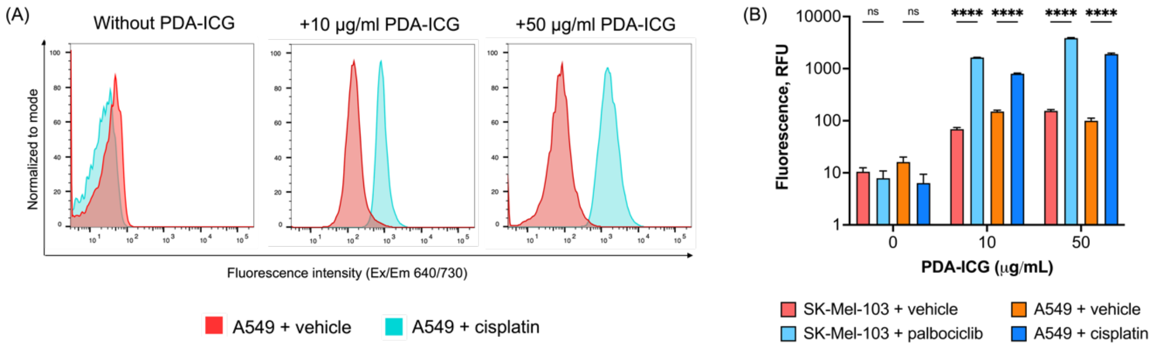

Figure S18. **Additional result for cellular uptake of PDA-ICG quantified using flow cytometry.** (A) Histogram showing mean fluorescence values of A549 cells after incubation with PDA-ICG; fluorescence was measured from the Alexa Fluor 700 channel that corresponded to the fluorescence of PDA-ICG. (B) Quantified geometric mean of fluorescence from the PDA-ICG channel of SK-Mel-103 and A539 cells after incubation with PDA-ICG conjugates. For both cell lines, drug-treated (senescent) cells showed higher fluorescence intensity, implying a higher uptake of PDA-ICG (mean ± s.d., n = 3).

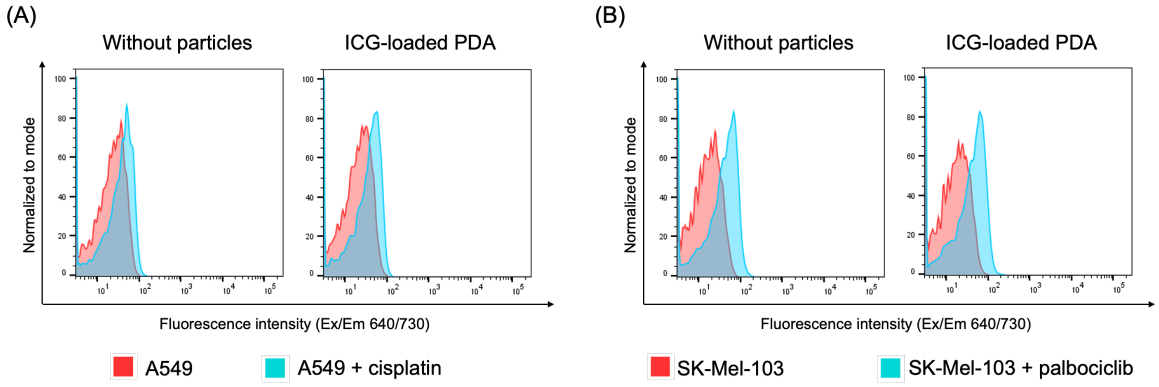

Figure S19. Flow cytometry quantifying uptake of ICG-loaded PDA in (A) A549 cells and (B) SK-Mel-103 cells. ICG-loaded PDA (prepared by simply mixing PDA with ICG at pH 3.0 without heating, see Figure S8B for the corresponding absorbance spectrum) showed lower overall cellular uptake, with similar uptake in both senescent and non-senescent cells, on the contrary to PDA-ICG nanoprobes.

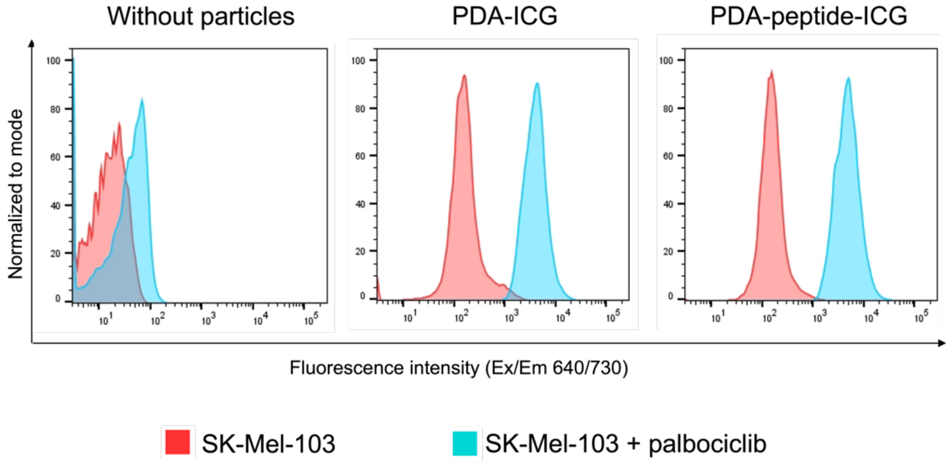

Figure S20. Flow cytometry quantifying uptake of PDA-peptide-ICG vs PDA-ICG by senescent and non-senescent SK-Mel-103 cells.

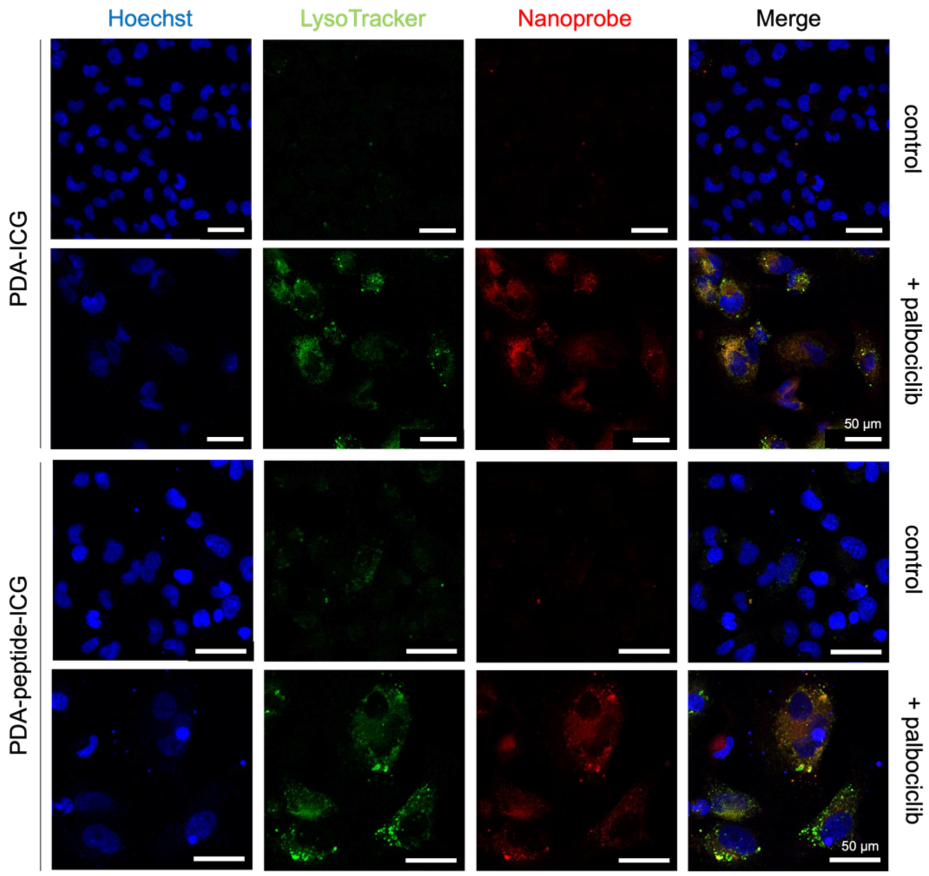

Figure S21. Fluorescence confocal images of SK-Mel-103 cells after incubation with PDA-ICG and PDA-peptide-ICG for 24 h. Cells were incubated with Hoechst 33342 (blue) and LysoTracker Green (green) to stain nuclei and lysosome. Alexa Fluor 700 channel (red) captured the fluorescence of PDA-ICG and PDA-peptide-ICG.

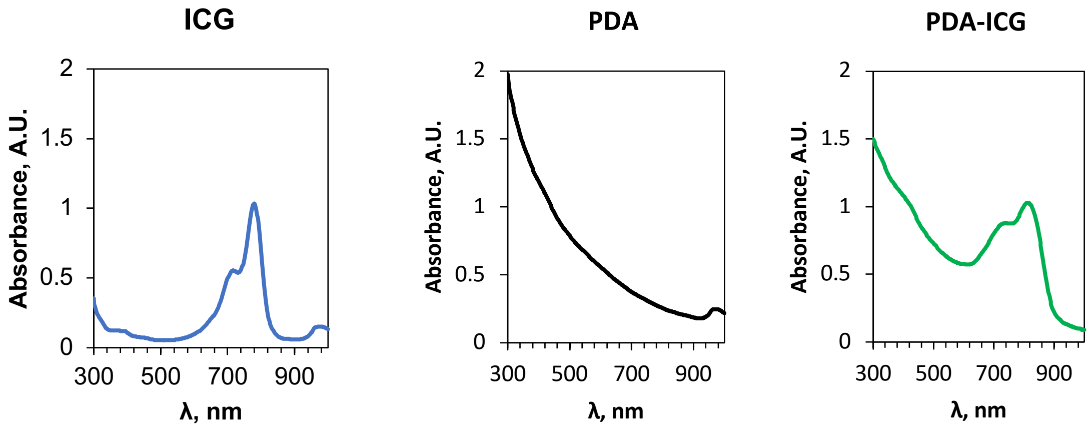

Figure S22. Absorbance of ICG, PDA and PDA-ICG for PAT measurements.

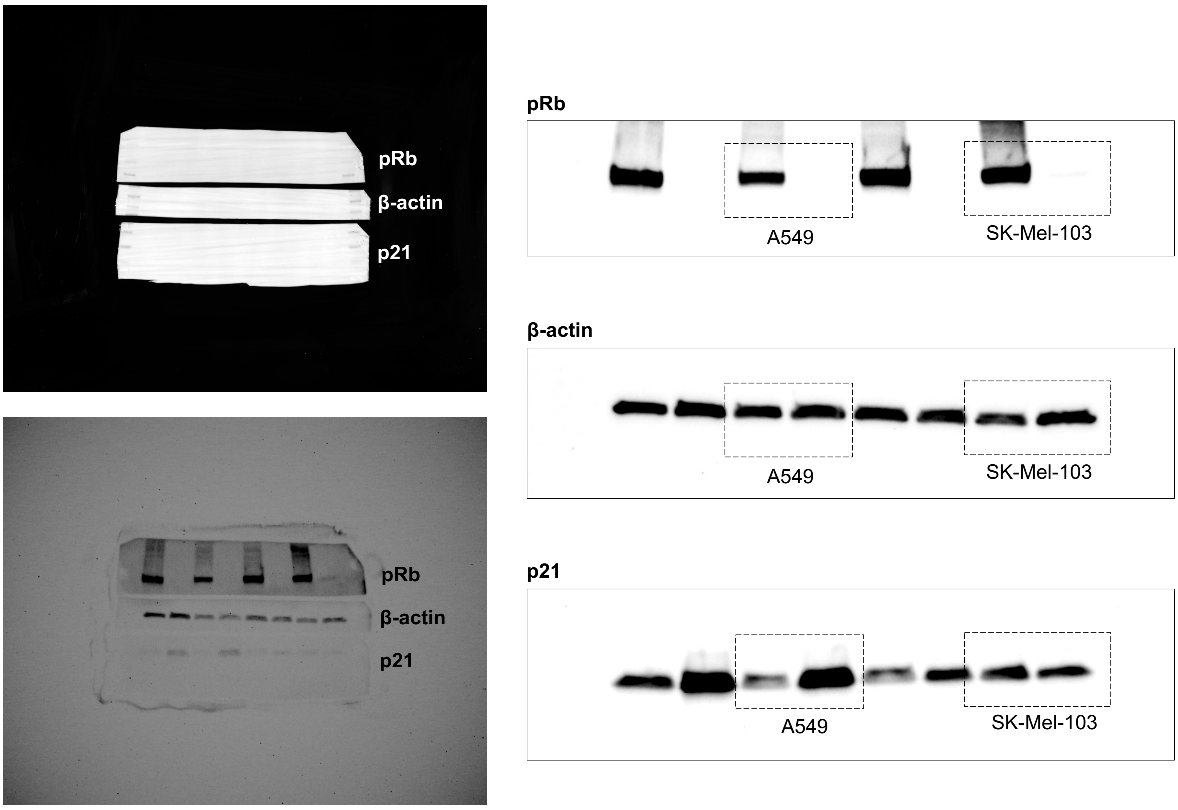

Figure S23. Uncropped western blot shown in Figure 4c.

Table S1. DLS and zeta potential measurement of products after mixing PDA and ICG in various conditions.

Reaction T, ºC	Samples	Size [nm]	Zeta potential [mV]
65	PDA bare	158 ± 52	-14 ± 6
	PDA after mixing with ICG at pH 6.2	150 ± 41	-13 ± 6
	PDA after mixing with ICG at pH 8.0	152 ± 52	-14 ± 3
25	PDA after mixing with ICG at pH 3.0	160 ± 43	-12 ± 3
	PDA after mixing with ICG in deionised water	154 ± 19	-15 ± 4

Table S2. Elemental CHN combustion results of PDA, PDA-arginine and PDA-arginine-ICG.

Samples	%C	%H	%N
PDA	53.79	3.37	7.43
PDA-arginine	55.79	4.49	10.90
PDA-arginine-ICG	60.64	4.90	16.36

Table S3. List of primers used to amplify senescence-associated target genes during RT-qPCR.

Target gene	Forward (5' – 3')	Reverse (5'-3')
ACTB	AGAAGGATTCCTATGTGGGC	TACTTCAGGGTGAGGATGC
LMNB1	GTATGAAGAGGAGATTAACGAGAC	TACTCAATTTGACGCCCAG
CDKN1A	CAGCATGACAGATTTCTACC	CAGGGTATGTACATGAGGAG
GLB1	GACAGTACCAGTTTTCTGAG	ATAGACTCTTTCTCTAGCAGC

Table S4. List of primary antibodies used for Western blot.

Antibody	Host species	Company	Catalogue no.	Working dilution
Anti-β-actin	Rabbit	Proteintech	20536-1-AP	1:1000
Anti-pRb	Rabbit	Cell signaling	D20B12	1:4000
Anti-p21	Rabbit	Abcam	ab109520	1:1000

Table S5. List of secondary antibodies used for Western blot.

Antibody	Host species	Company	Catalogue no.	Working dilution
HRP-Conjugated AffiniPute Anti-Rabbit IgG	Donkey	Jackson ImmunoResearch	711-035-152	1:5000
